# Supplementary material for: CHIKV infection reprograms codon optimality to favor viral RNA translation by altering the tRNA epitranscriptome
Source: Nat Commun. 2022 Aug 11;13:4725. doi: 10.1038/s41467-022-31835-x (PMC9366759; doi:10.1038/s41467-022-31835-x)
Supplement: Supplementary file 1 — Supplementary Information [file 41467_2022_31835_MOESM1_ESM.pptx]

## Slide 1
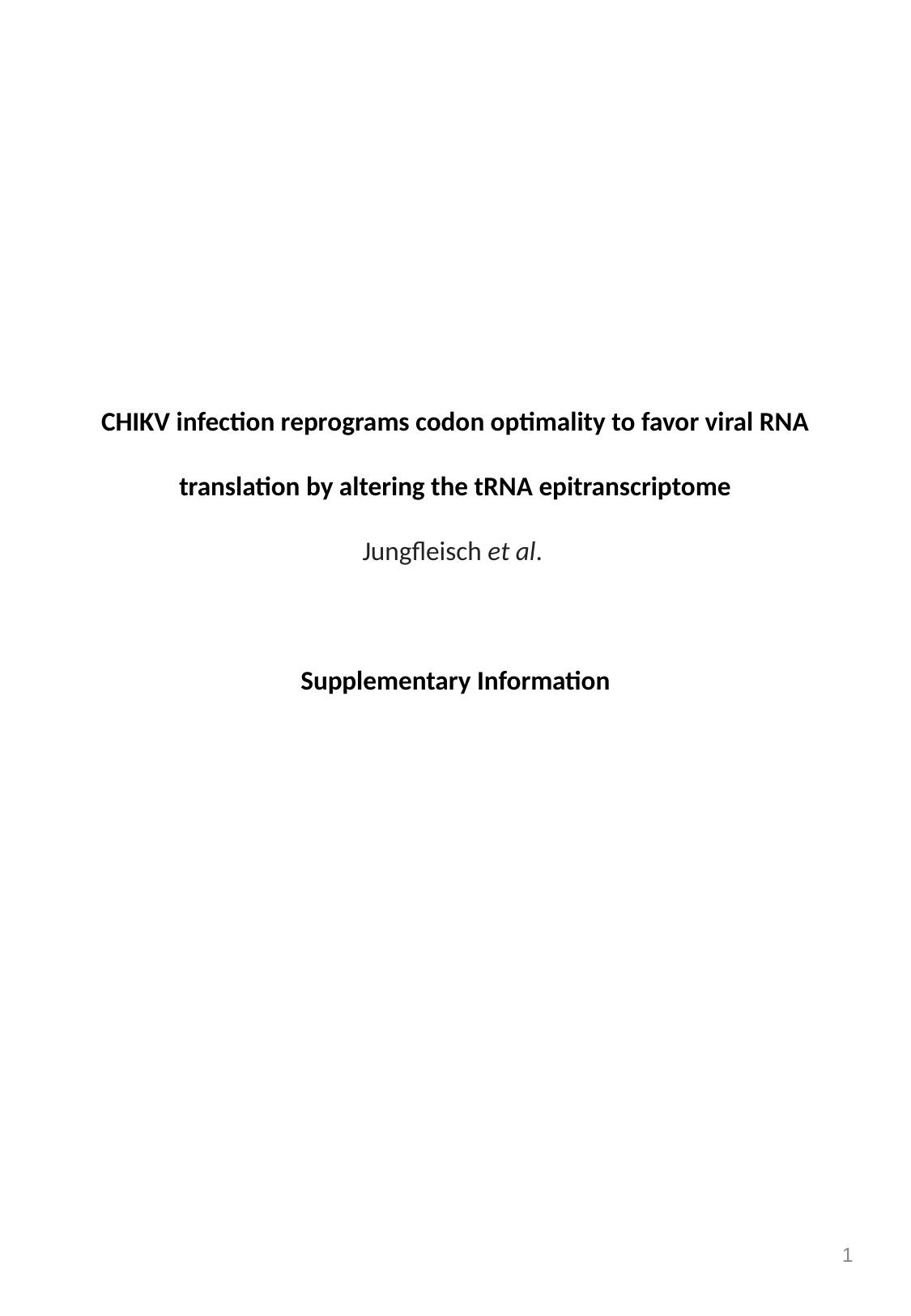

# CHIKV infection reprograms codon optimality to favor viral RNA translation by altering the tRNA epitranscriptome Jungfleisch et al.   Supplementary Information
1

## Slide 2
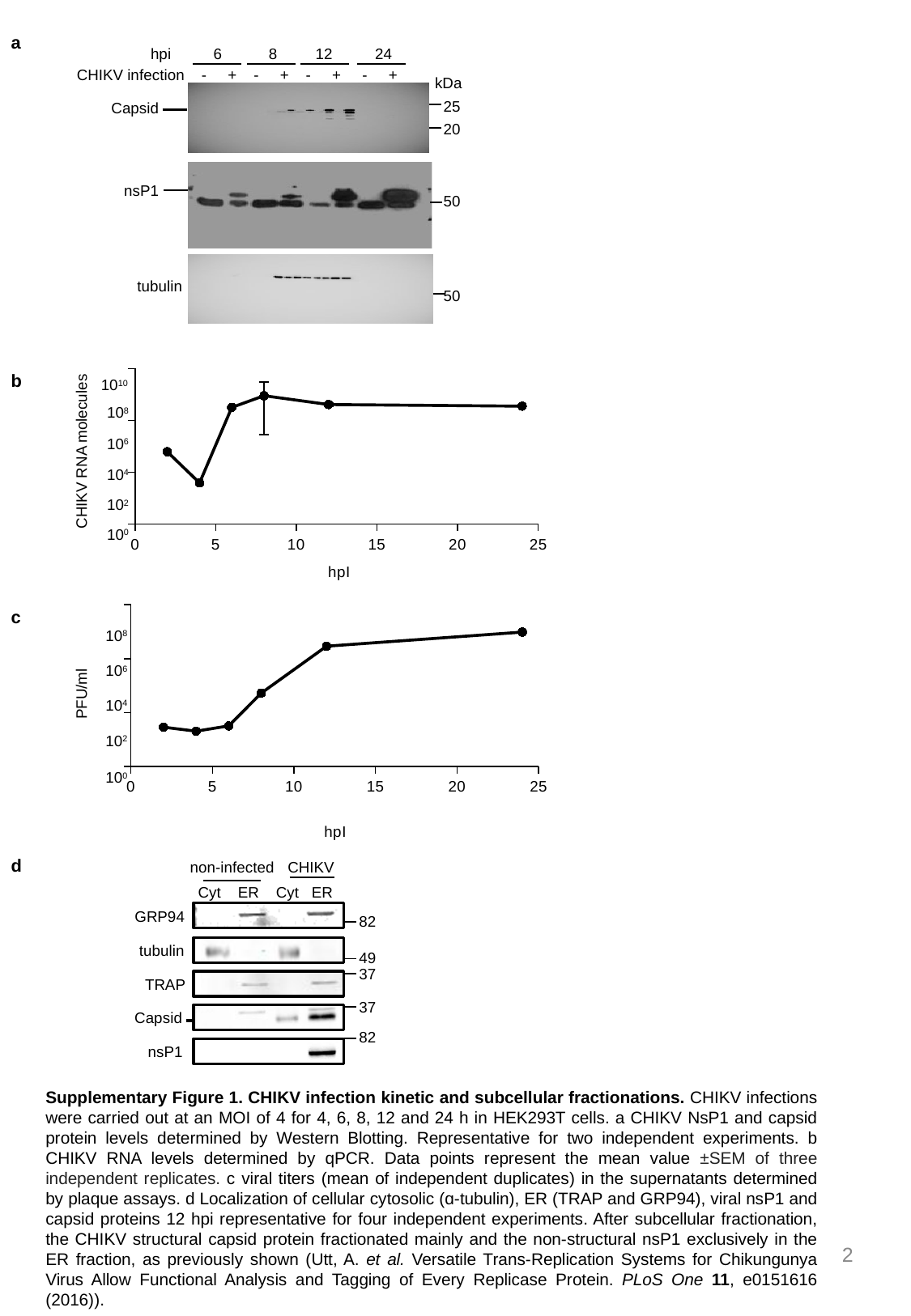

a
 hpi 6 8 12 24
 CHIKV infection - + - + - + - +
kDa
25
Capsid
20
nsP1
50
tubulin
50
b
### Chart
| Category | |
|---|---|1010
108
CHIKV RNA molecules
106
104
102
100
c
### Chart
| Category | |
|---|---|108
PFU/ml
106
104
102
100
d
non-infected
CHIKV
Cyt ER Cyt ER
GRP94
tubulin
TRAP
Capsid
nsP1
82
49
37
37
82
Supplementary Figure 1. CHIKV infection kinetic and subcellular fractionations. CHIKV infections were carried out at an MOI of 4 for 4, 6, 8, 12 and 24 h in HEK293T cells. a CHIKV NsP1 and capsid protein levels determined by Western Blotting. Representative for two independent experiments. b CHIKV RNA levels determined by qPCR. Data points represent the mean value ±SEM of three independent replicates. c viral titers (mean of independent duplicates) in the supernatants determined by plaque assays. d Localization of cellular cytosolic (ɑ-tubulin), ER (TRAP and GRP94), viral nsP1 and capsid proteins 12 hpi representative for four independent experiments. After subcellular fractionation, the CHIKV structural capsid protein fractionated mainly and the non-structural nsP1 exclusively in the ER fraction, as previously shown (Utt, A. et al. Versatile Trans-Replication Systems for Chikungunya Virus Allow Functional Analysis and Tagging of Every Replicase Protein. PLoS One 11, e0151616 (2016)).
2

## Slide 3
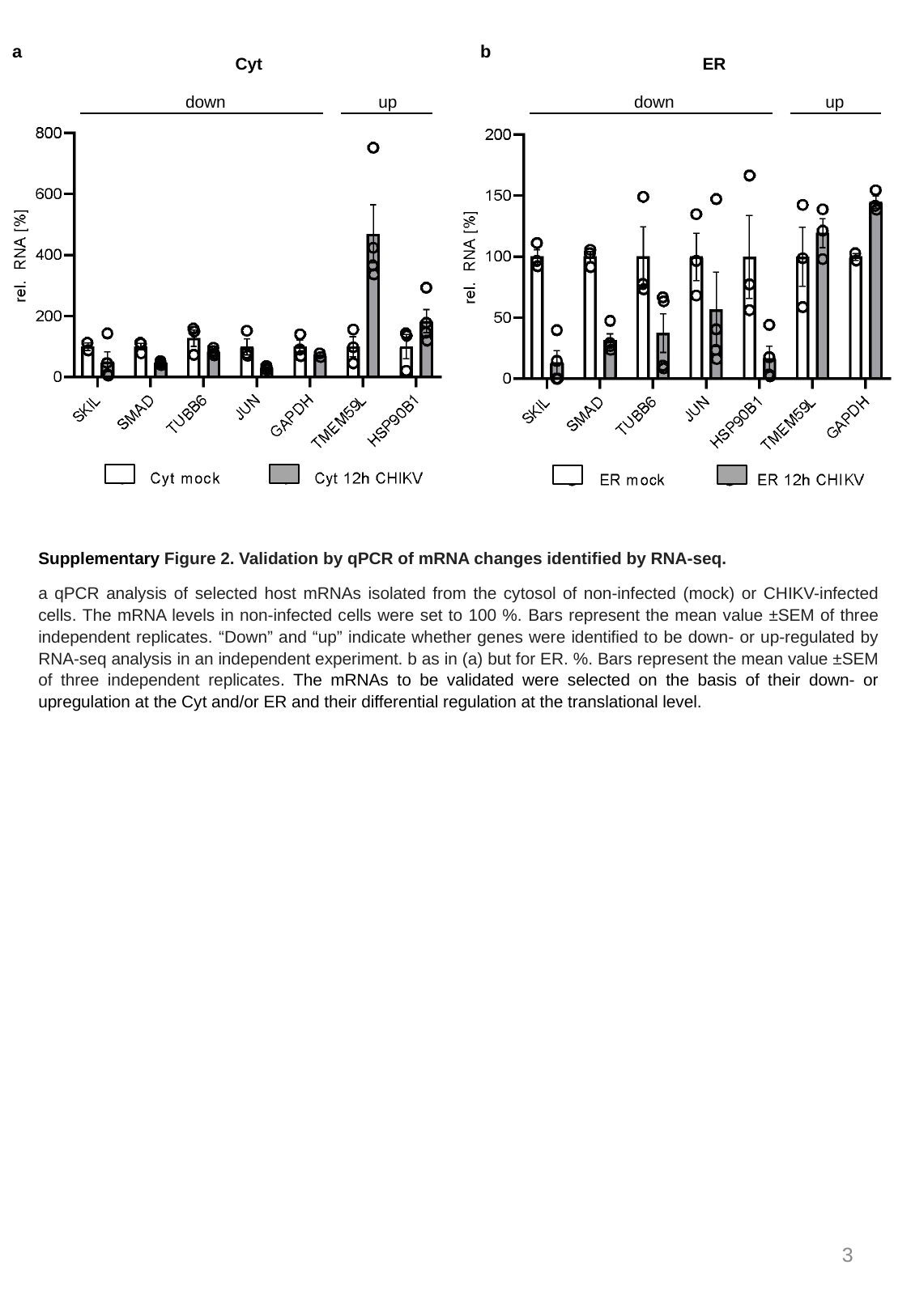

a
b
Cyt
ER
down
up
down
up
Supplementary Figure 2. Validation by qPCR of mRNA changes identified by RNA-seq.
a qPCR analysis of selected host mRNAs isolated from the cytosol of non-infected (mock) or CHIKV-infected cells. The mRNA levels in non-infected cells were set to 100 %. Bars represent the mean value ±SEM of three independent replicates. “Down” and “up” indicate whether genes were identified to be down- or up-regulated by RNA-seq analysis in an independent experiment. b as in (a) but for ER. %. Bars represent the mean value ±SEM of three independent replicates. The mRNAs to be validated were selected on the basis of their down- or upregulation at the Cyt and/or ER and their differential regulation at the translational level.
3

## Slide 4
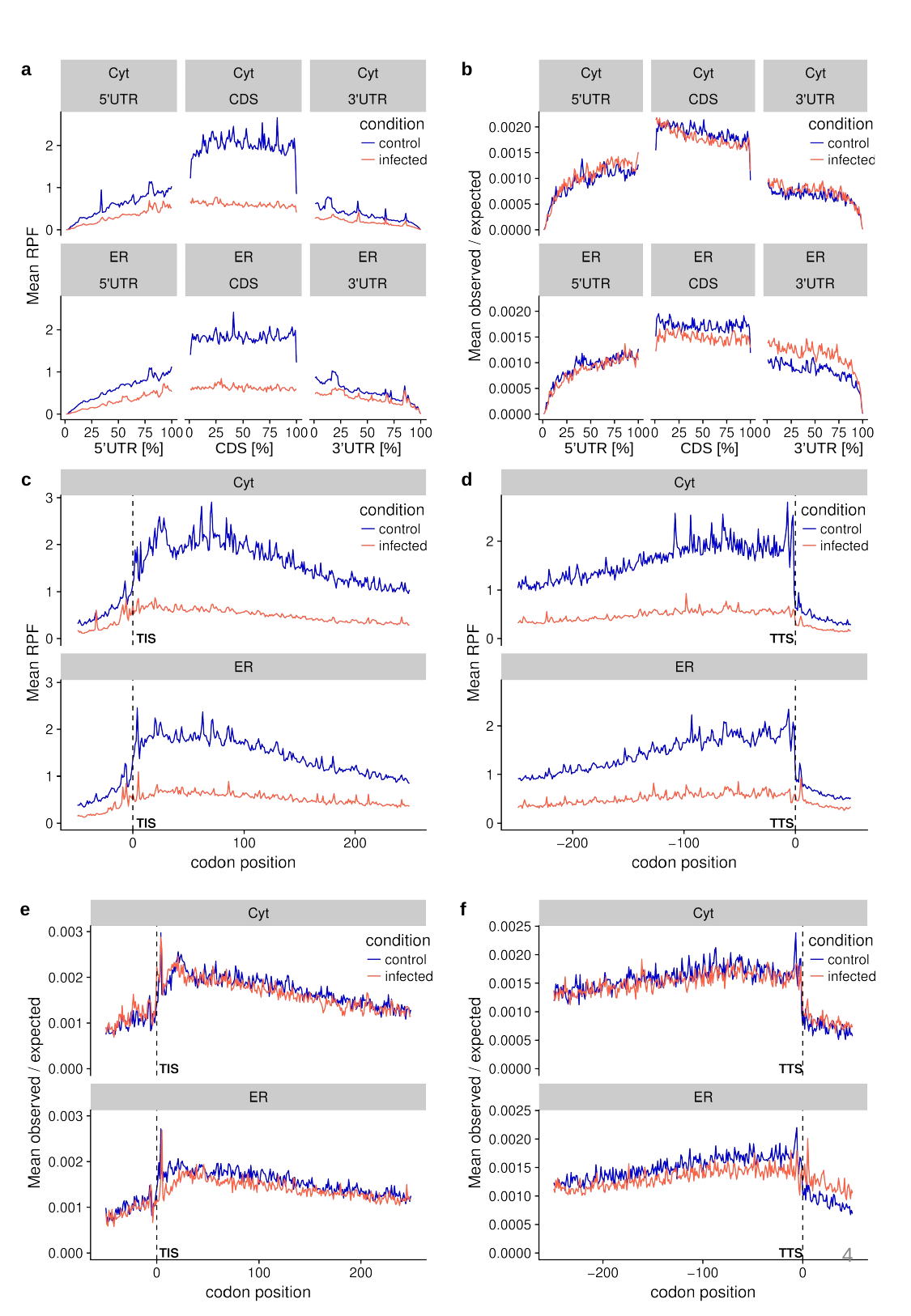

a
b
5’UTR [%]
CDS [%]
3’UTR [%]
5’UTR [%]
CDS [%]
3’UTR [%]
c
d
e
f
4

## Slide 5
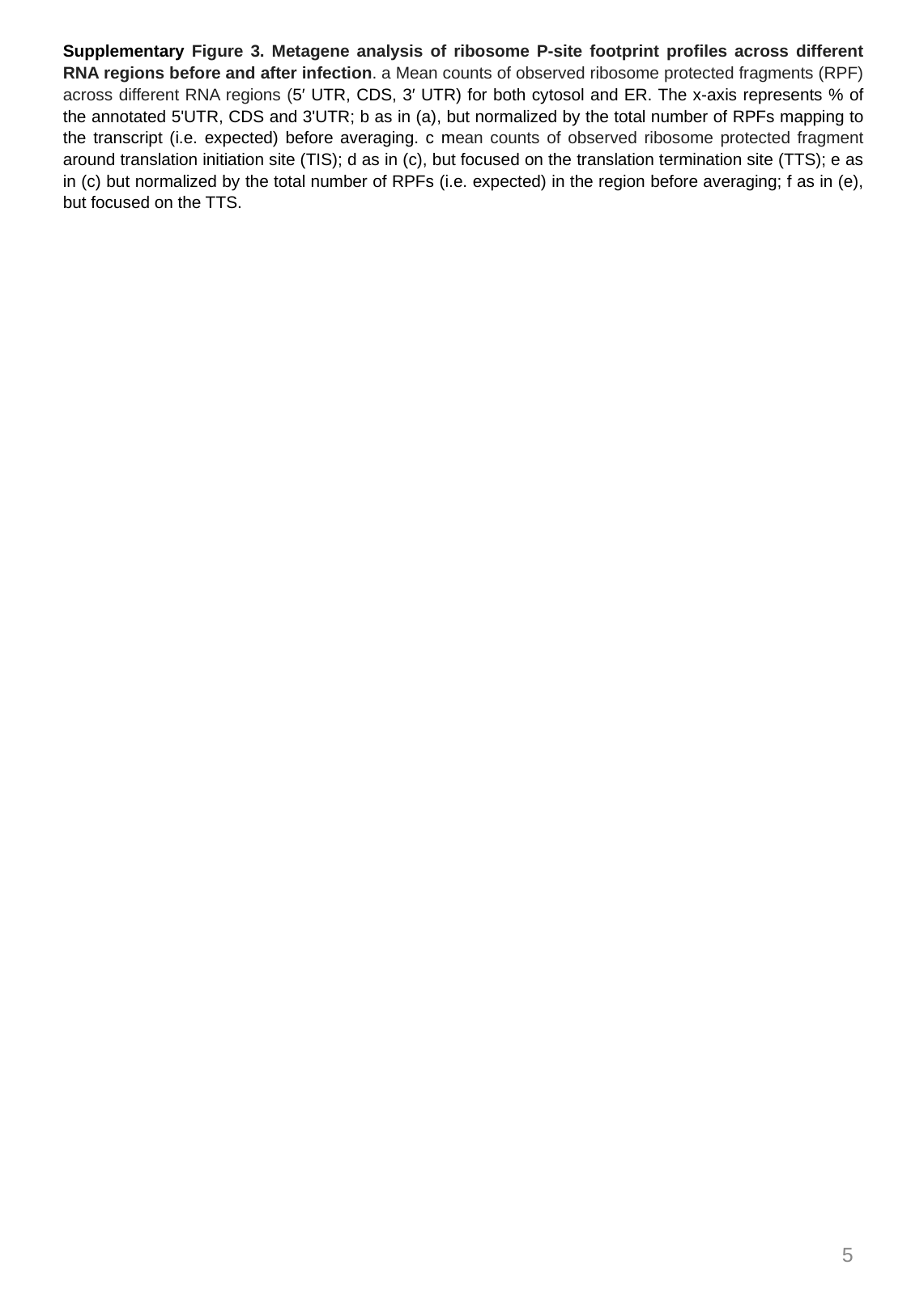

Supplementary Figure 3. Metagene analysis of ribosome P-site footprint profiles across different RNA regions before and after infection. a Mean counts of observed ribosome protected fragments (RPF) across different RNA regions (5′ UTR, CDS, 3′ UTR) for both cytosol and ER. The x-axis represents % of the annotated 5'UTR, CDS and 3'UTR; b as in (a), but normalized by the total number of RPFs mapping to the transcript (i.e. expected) before averaging. c mean counts of observed ribosome protected fragment around translation initiation site (TIS); d as in (c), but focused on the translation termination site (TTS); e as in (c) but normalized by the total number of RPFs (i.e. expected) in the region before averaging; f as in (e), but focused on the TTS.
5

## Slide 6
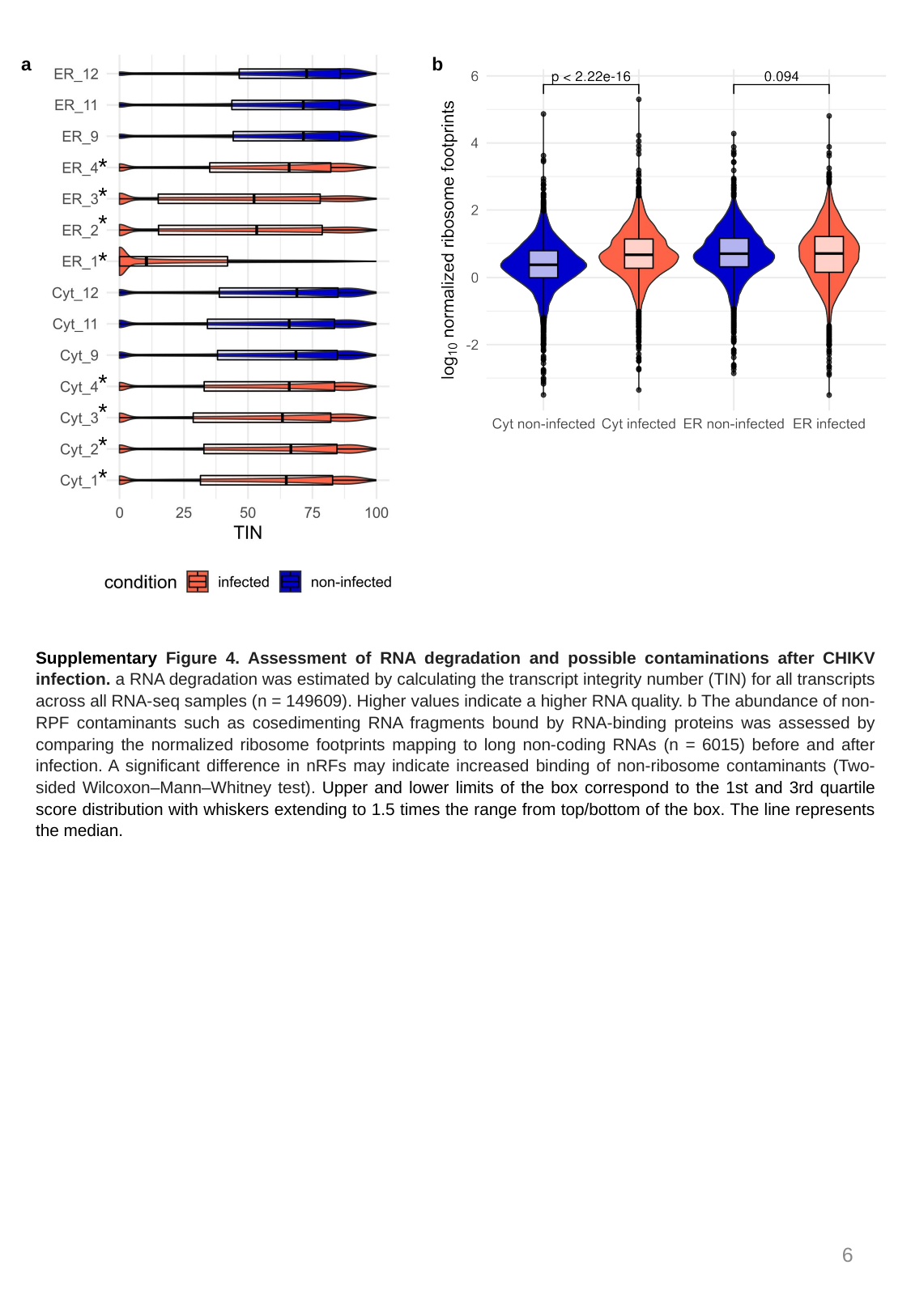

a
b
*
*
*
*
*
*
*
*
Supplementary Figure 4. Assessment of RNA degradation and possible contaminations after CHIKV infection. a RNA degradation was estimated by calculating the transcript integrity number (TIN) for all transcripts across all RNA-seq samples (n = 149609). Higher values indicate a higher RNA quality. b The abundance of non-RPF contaminants such as cosedimenting RNA fragments bound by RNA-binding proteins was assessed by comparing the normalized ribosome footprints mapping to long non-coding RNAs (n = 6015) before and after infection. A significant difference in nRFs may indicate increased binding of non-ribosome contaminants (Two-sided Wilcoxon–Mann–Whitney test). Upper and lower limits of the box correspond to the 1st and 3rd quartile score distribution with whiskers extending to 1.5 times the range from top/bottom of the box. The line represents the median.
6

## Slide 7
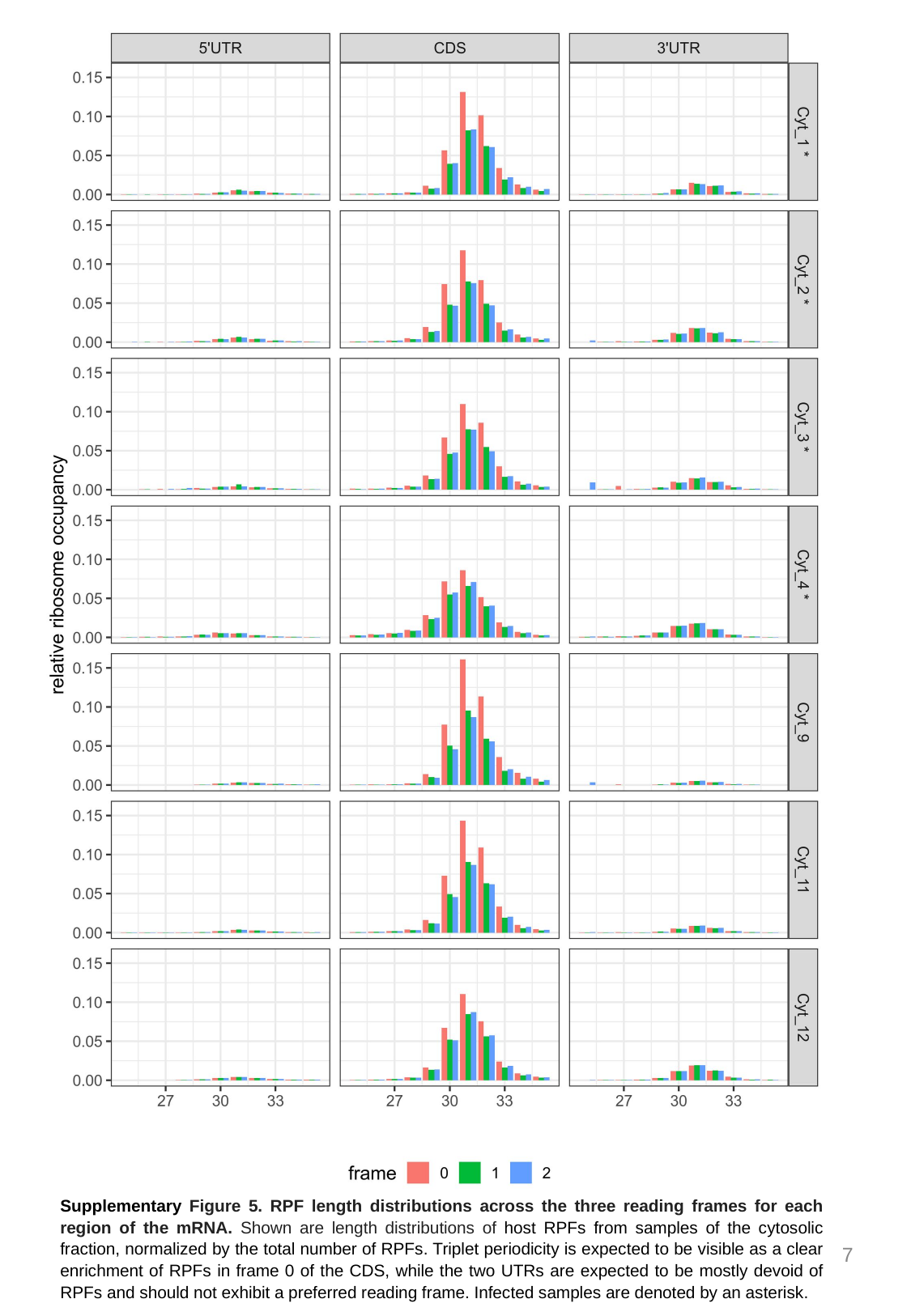

Supplementary Figure 5. RPF length distributions across the three reading frames for each region of the mRNA. Shown are length distributions of host RPFs from samples of the cytosolic fraction, normalized by the total number of RPFs. Triplet periodicity is expected to be visible as a clear enrichment of RPFs in frame 0 of the CDS, while the two UTRs are expected to be mostly devoid of RPFs and should not exhibit a preferred reading frame. Infected samples are denoted by an asterisk.
7

## Slide 8
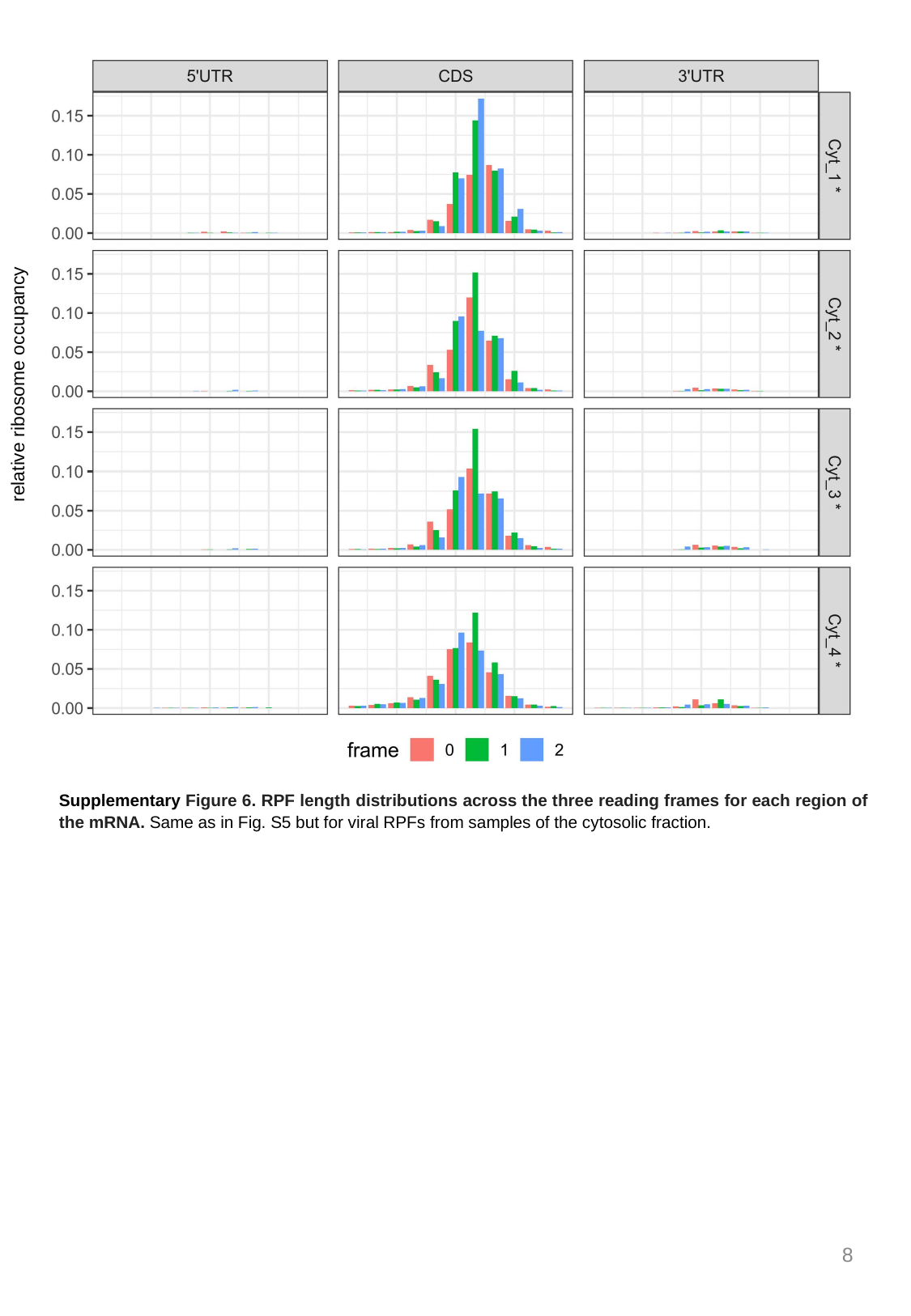

relative ribosome occupancy
Supplementary Figure 6. RPF length distributions across the three reading frames for each region of the mRNA. Same as in Fig. S5 but for viral RPFs from samples of the cytosolic fraction.
8

## Slide 9
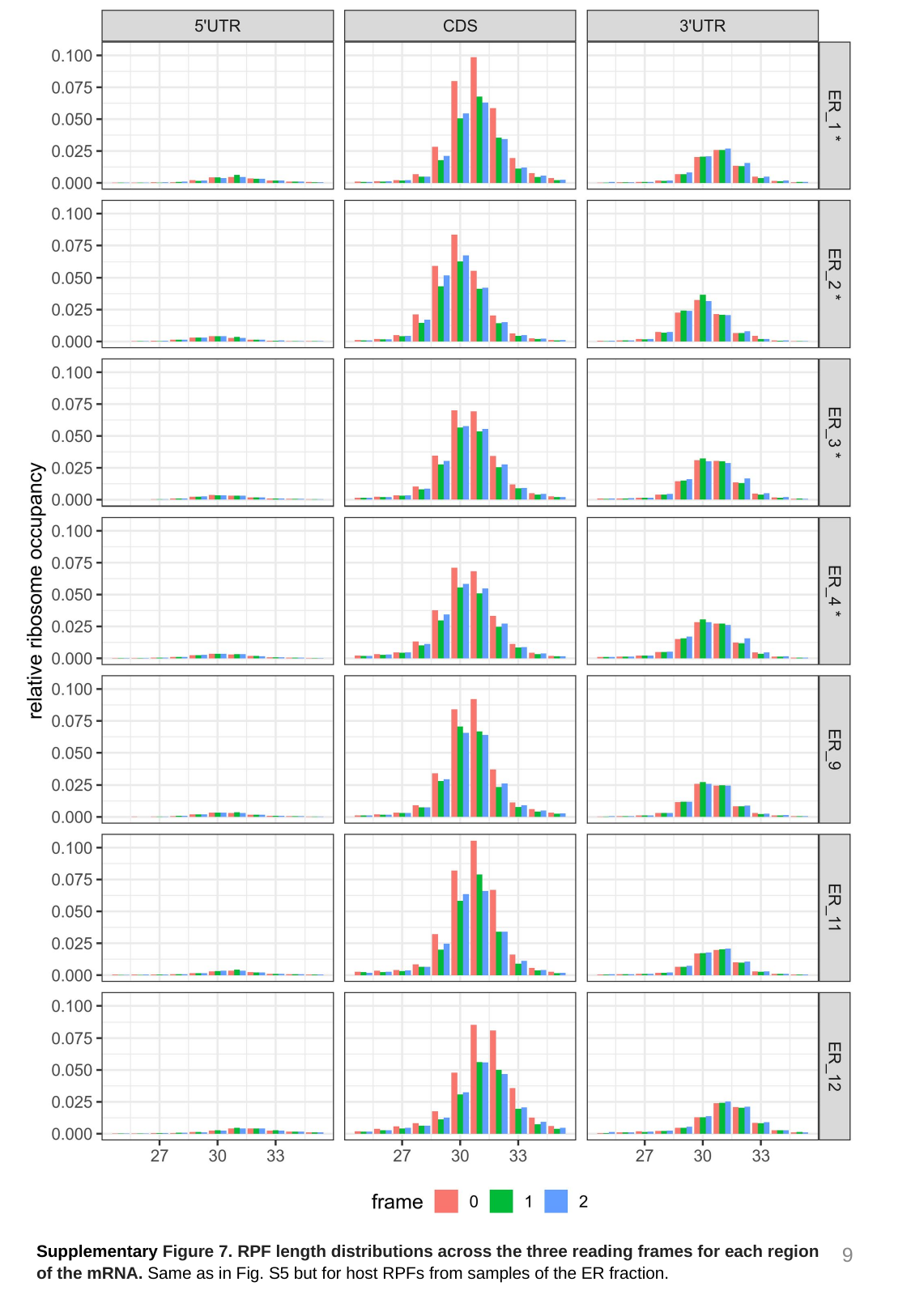

9
Supplementary Figure 7. RPF length distributions across the three reading frames for each region of the mRNA. Same as in Fig. S5 but for host RPFs from samples of the ER fraction.

## Slide 10
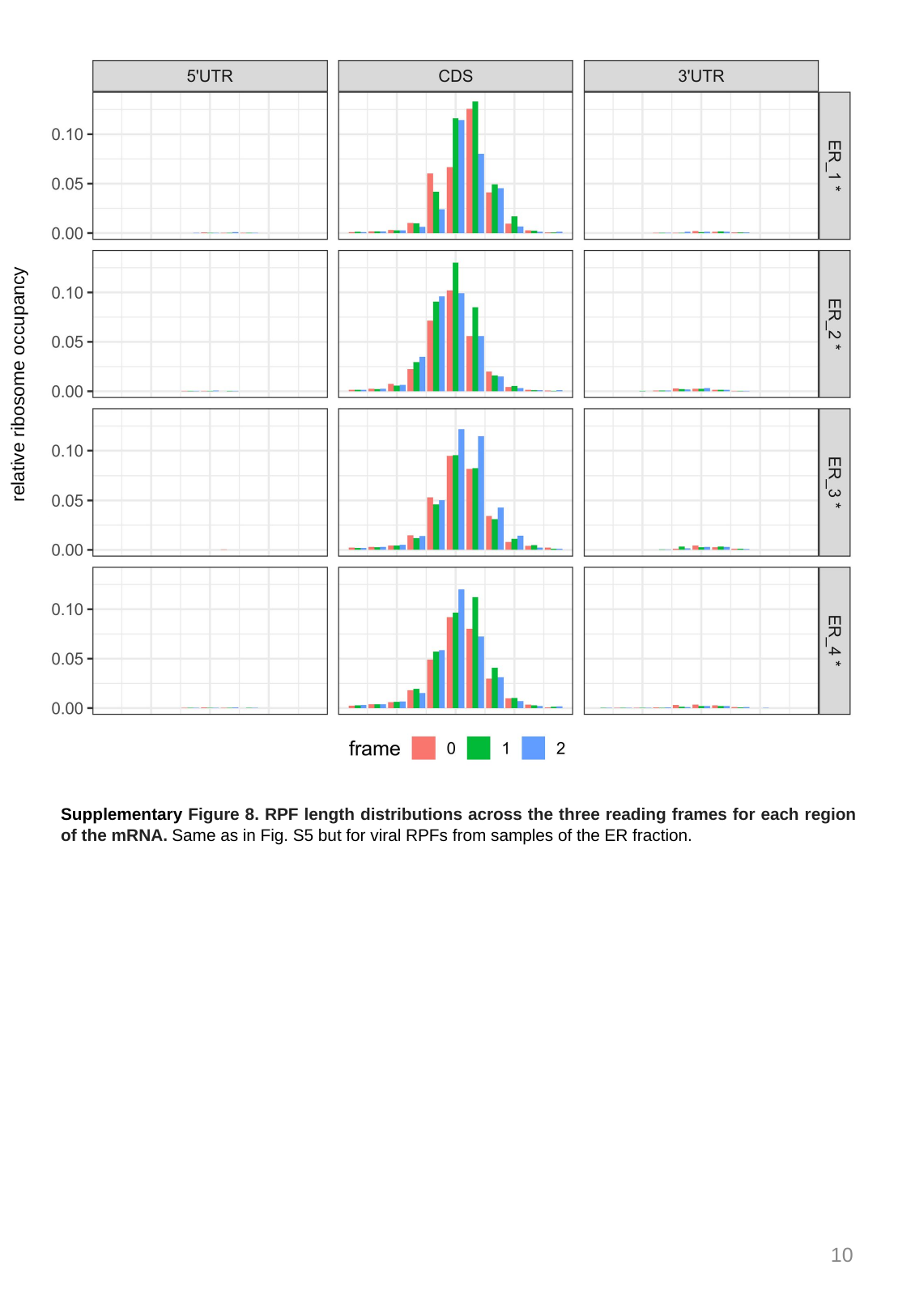

relative ribosome occupancy
Supplementary Figure 8. RPF length distributions across the three reading frames for each region of the mRNA. Same as in Fig. S5 but for viral RPFs from samples of the ER fraction.
10

## Slide 11
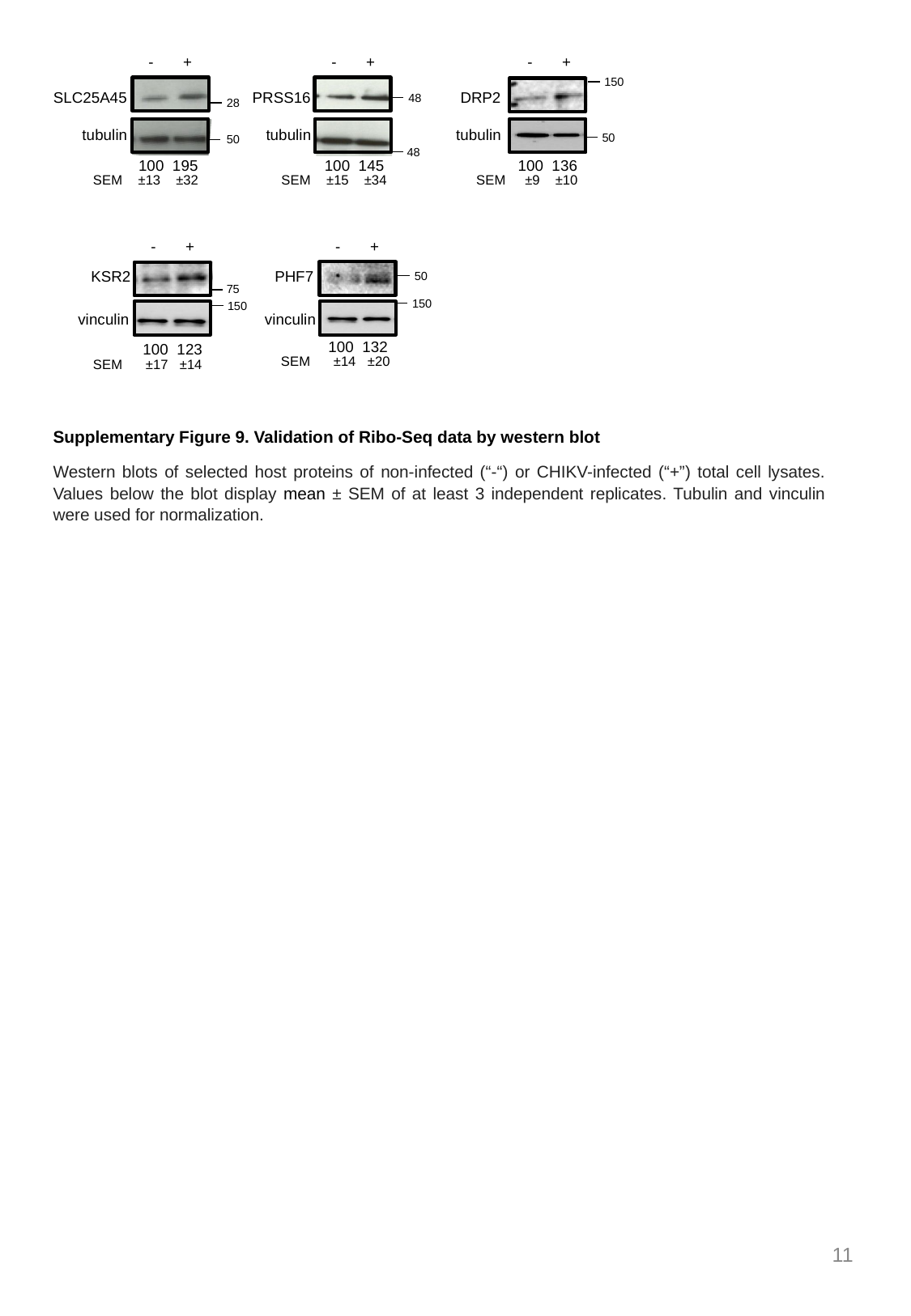

- +
- +
- +
150
SLC25A45
PRSS16
DRP2
48
28
tubulin
tubulin
tubulin
50
50
48
100 195
100 145
100 136
 SEM ±13 ±32
 SEM ±15 ±34
 SEM ±9 ±10
- +
- +
KSR2
PHF7
50
75
150
150
vinculin
vinculin
100 132
100 123
 SEM ±14 ±20
 SEM ±17 ±14
Supplementary Figure 9. Validation of Ribo-Seq data by western blot
Western blots of selected host proteins of non-infected (“-“) or CHIKV-infected (“+”) total cell lysates. Values below the blot display mean ± SEM of at least 3 independent replicates. Tubulin and vinculin were used for normalization.
11

## Slide 12
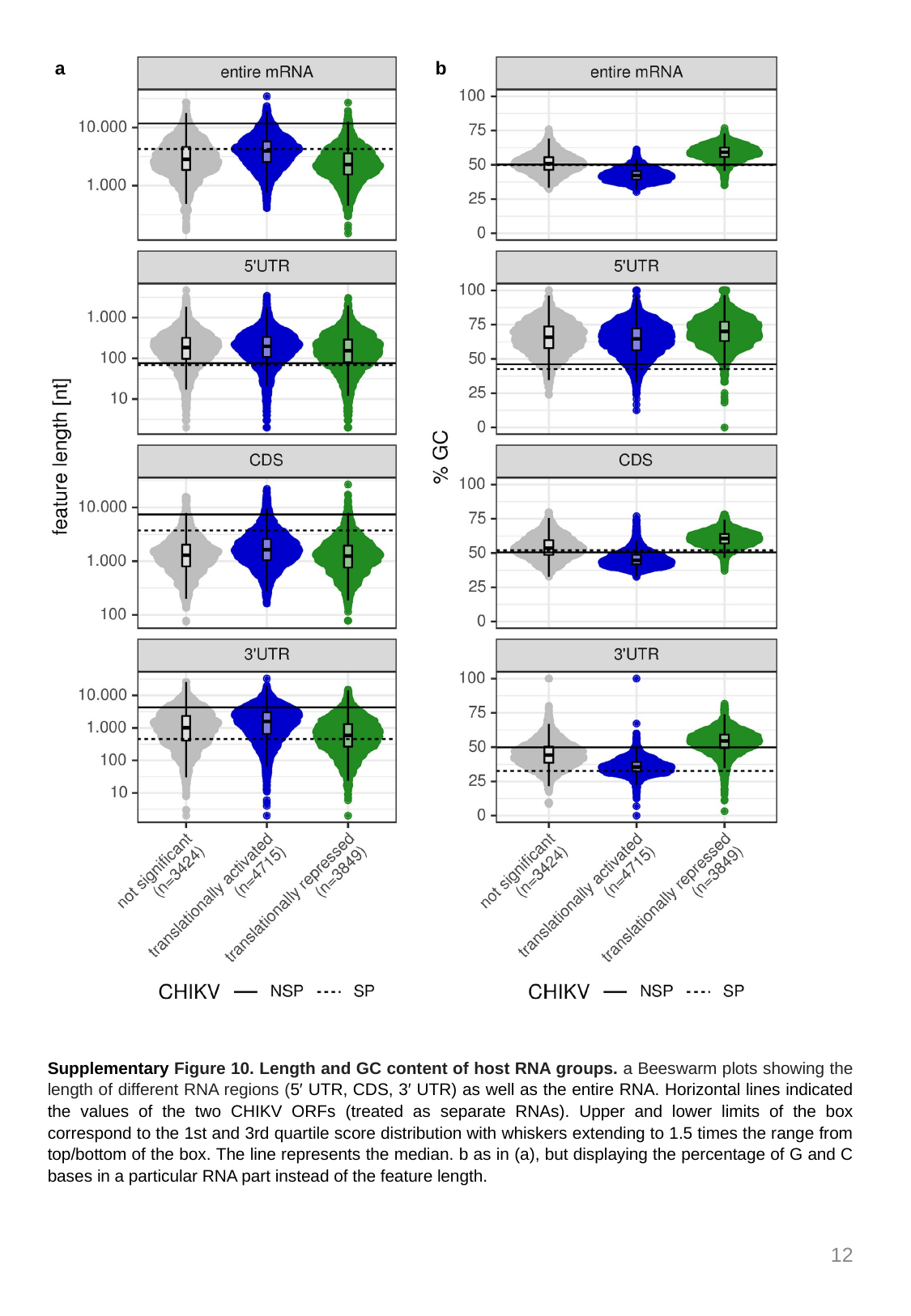

a
b
Supplementary Figure 10. Length and GC content of host RNA groups. a Beeswarm plots showing the length of different RNA regions (5′ UTR, CDS, 3′ UTR) as well as the entire RNA. Horizontal lines indicated the values of the two CHIKV ORFs (treated as separate RNAs). Upper and lower limits of the box correspond to the 1st and 3rd quartile score distribution with whiskers extending to 1.5 times the range from top/bottom of the box. The line represents the median. b as in (a), but displaying the percentage of G and C bases in a particular RNA part instead of the feature length.
12

## Slide 13
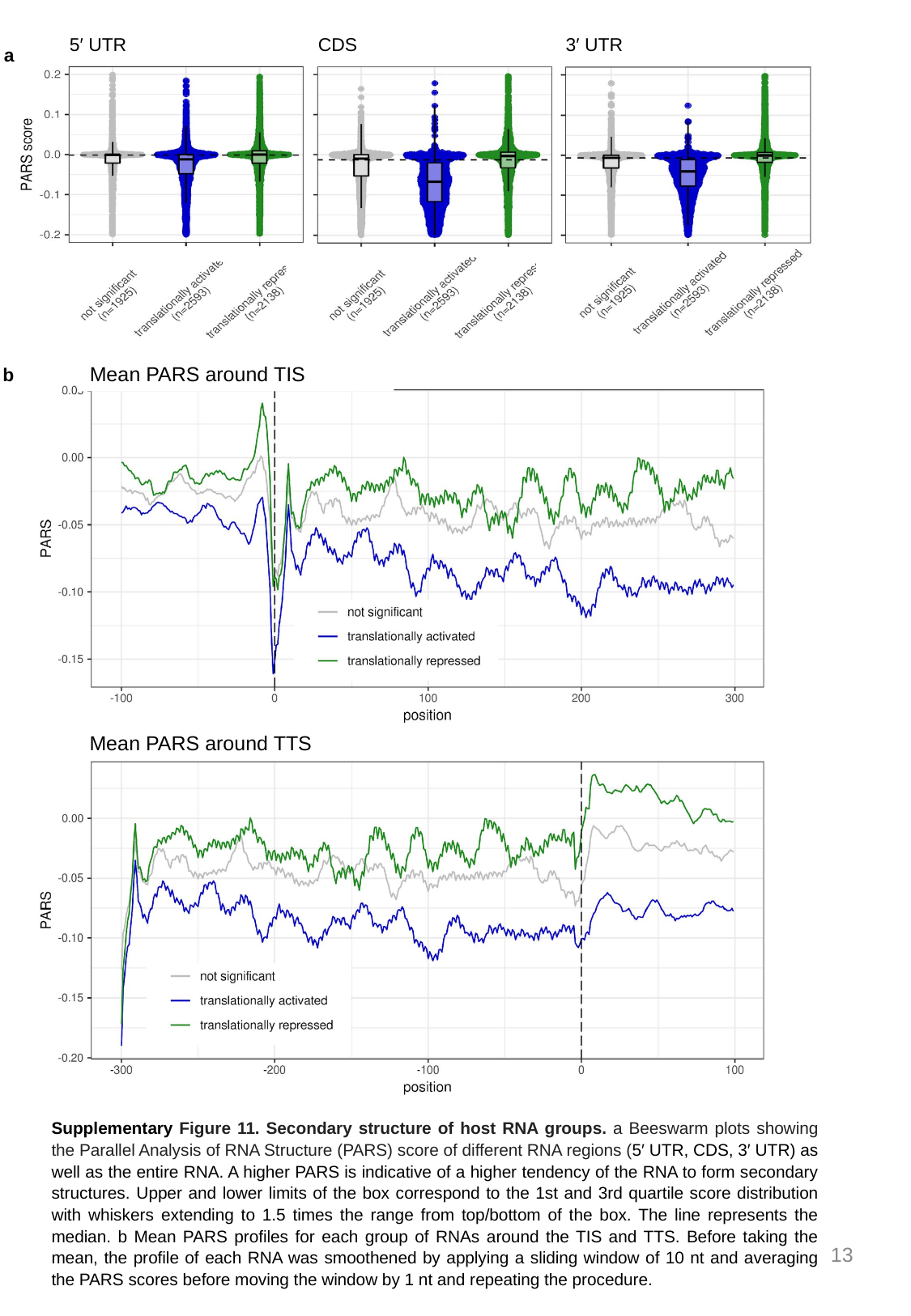

5′ UTR
CDS
3′ UTR
a
Mean PARS around TIS
b
Mean PARS around TTS
Supplementary Figure 11. Secondary structure of host RNA groups. a Beeswarm plots showing the Parallel Analysis of RNA Structure (PARS) score of different RNA regions (5′ UTR, CDS, 3′ UTR) as well as the entire RNA. A higher PARS is indicative of a higher tendency of the RNA to form secondary structures. Upper and lower limits of the box correspond to the 1st and 3rd quartile score distribution with whiskers extending to 1.5 times the range from top/bottom of the box. The line represents the median. b Mean PARS profiles for each group of RNAs around the TIS and TTS. Before taking the mean, the profile of each RNA was smoothened by applying a sliding window of 10 nt and averaging the PARS scores before moving the window by 1 nt and repeating the procedure.
13

## Slide 14
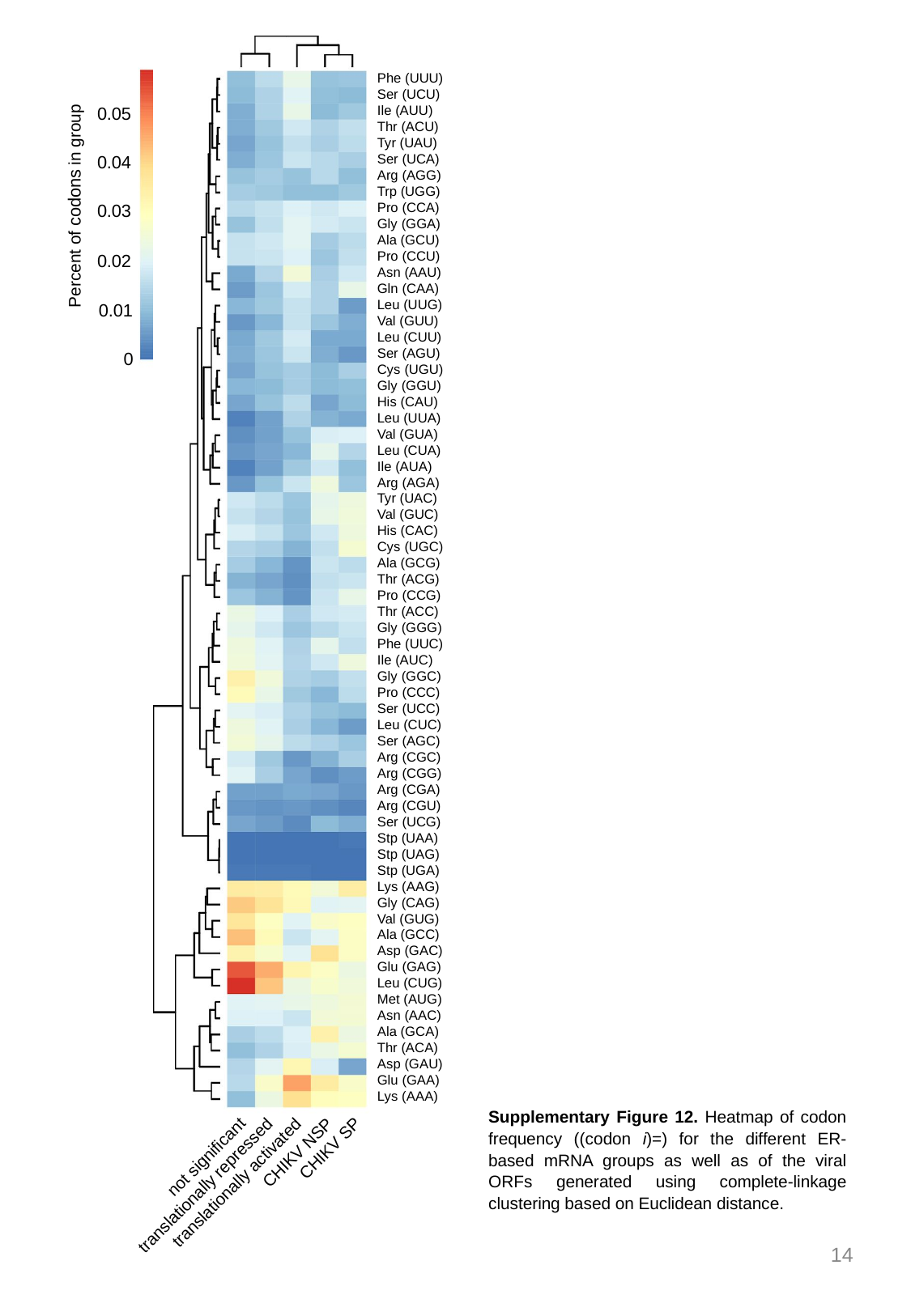

Phe (UUU)
Ser (UCU)
Ile (AUU)
Thr (ACU)
Tyr (UAU)
Ser (UCA)
Arg (AGG)
Trp (UGG)
Pro (CCA)
Gly (GGA)
Ala (GCU)
Pro (CCU)
Asn (AAU)
Gln (CAA)
Leu (UUG)
Val (GUU)
Leu (CUU)
Ser (AGU)
Cys (UGU)
Gly (GGU)
His (CAU)
Leu (UUA)
Val (GUA)
Leu (CUA)
Ile (AUA)
Arg (AGA)
Tyr (UAC)
Val (GUC)
His (CAC)
Cys (UGC)
Ala (GCG)
Thr (ACG)
Pro (CCG)
Thr (ACC)
Gly (GGG)
Phe (UUC)
Ile (AUC)
Gly (GGC)
Pro (CCC)
Ser (UCC)
Leu (CUC)
Ser (AGC)
Arg (CGC)
Arg (CGG)
Arg (CGA)
Arg (CGU)
Ser (UCG)
Stp (UAA)
Stp (UAG)
Stp (UGA)
Lys (AAG)
Gly (CAG)
Val (GUG)
Ala (GCC)
Asp (GAC)
Glu (GAG)
Leu (CUG)
Met (AUG)
Asn (AAC)
Ala (GCA)
Thr (ACA)
Asp (GAU)
Glu (GAA)
Lys (AAA)
0.05
0.04
0.03
0.02
0.01
0
Percent of codons in group
CHIKV SP
CHIKV NSP
not significant
translationally activated
translationally repressed
14

## Slide 15
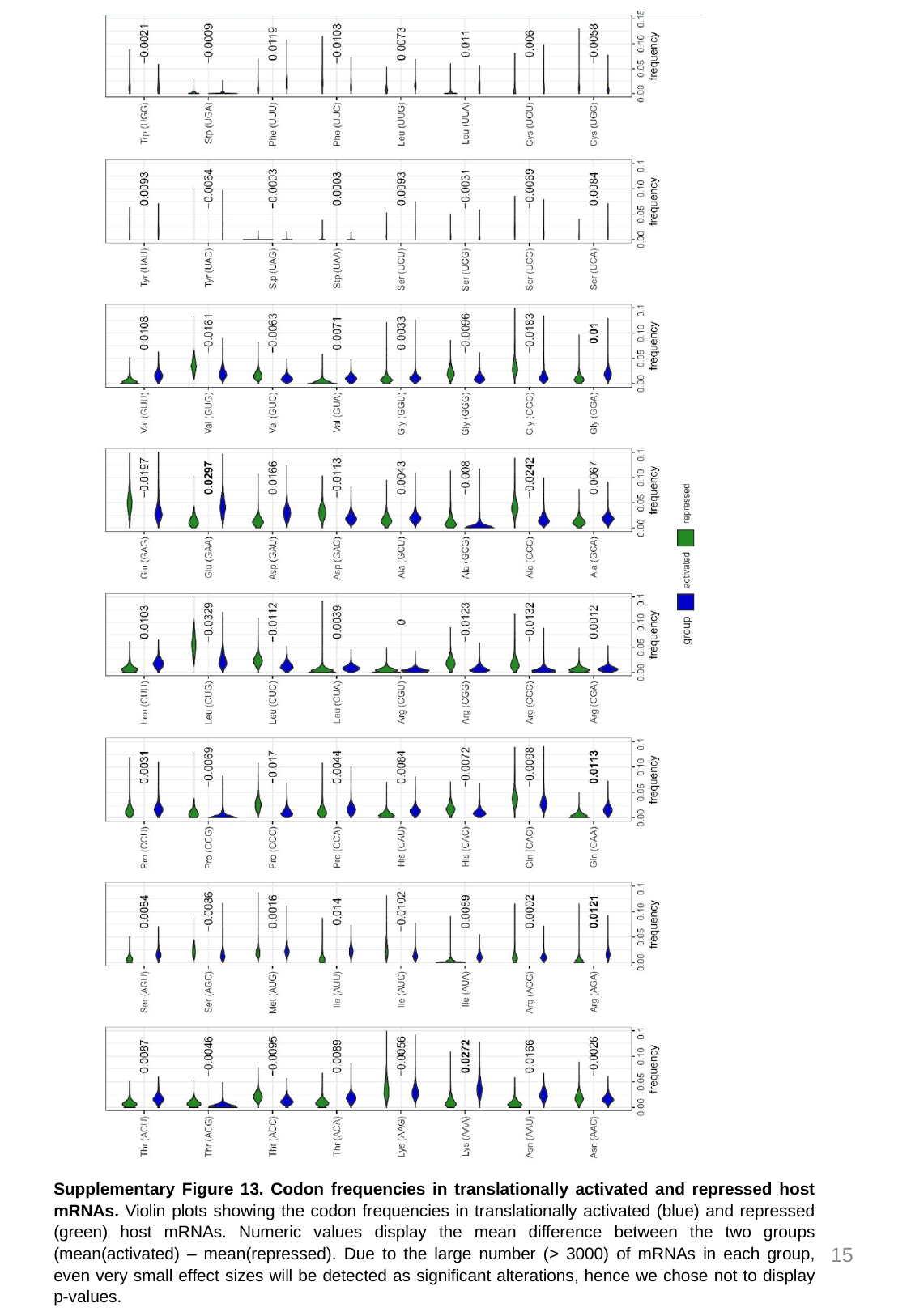

Supplementary Figure 13. Codon frequencies in translationally activated and repressed host mRNAs. Violin plots showing the codon frequencies in translationally activated (blue) and repressed (green) host mRNAs. Numeric values display the mean difference between the two groups (mean(activated) – mean(repressed). Due to the large number (> 3000) of mRNAs in each group, even very small effect sizes will be detected as significant alterations, hence we chose not to display p-values.
15

## Slide 16
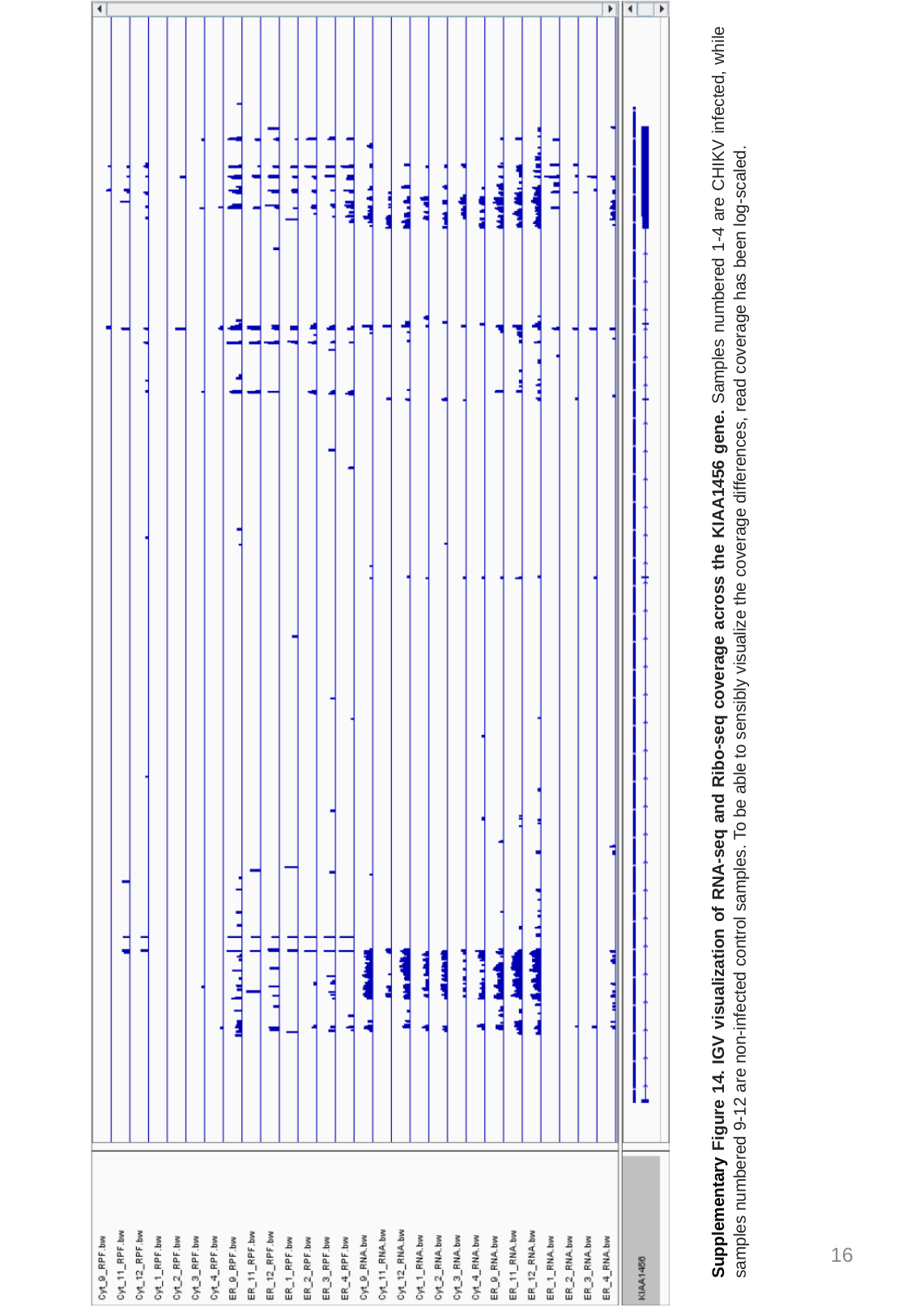

Supplementary Figure 14. IGV visualization of RNA-seq and Ribo-seq coverage across the KIAA1456 gene. Samples numbered 1-4 are CHIKV infected, while samples numbered 9-12 are non-infected control samples. To be able to sensibly visualize the coverage differences, read coverage has been log-scaled.
16

## Slide 17
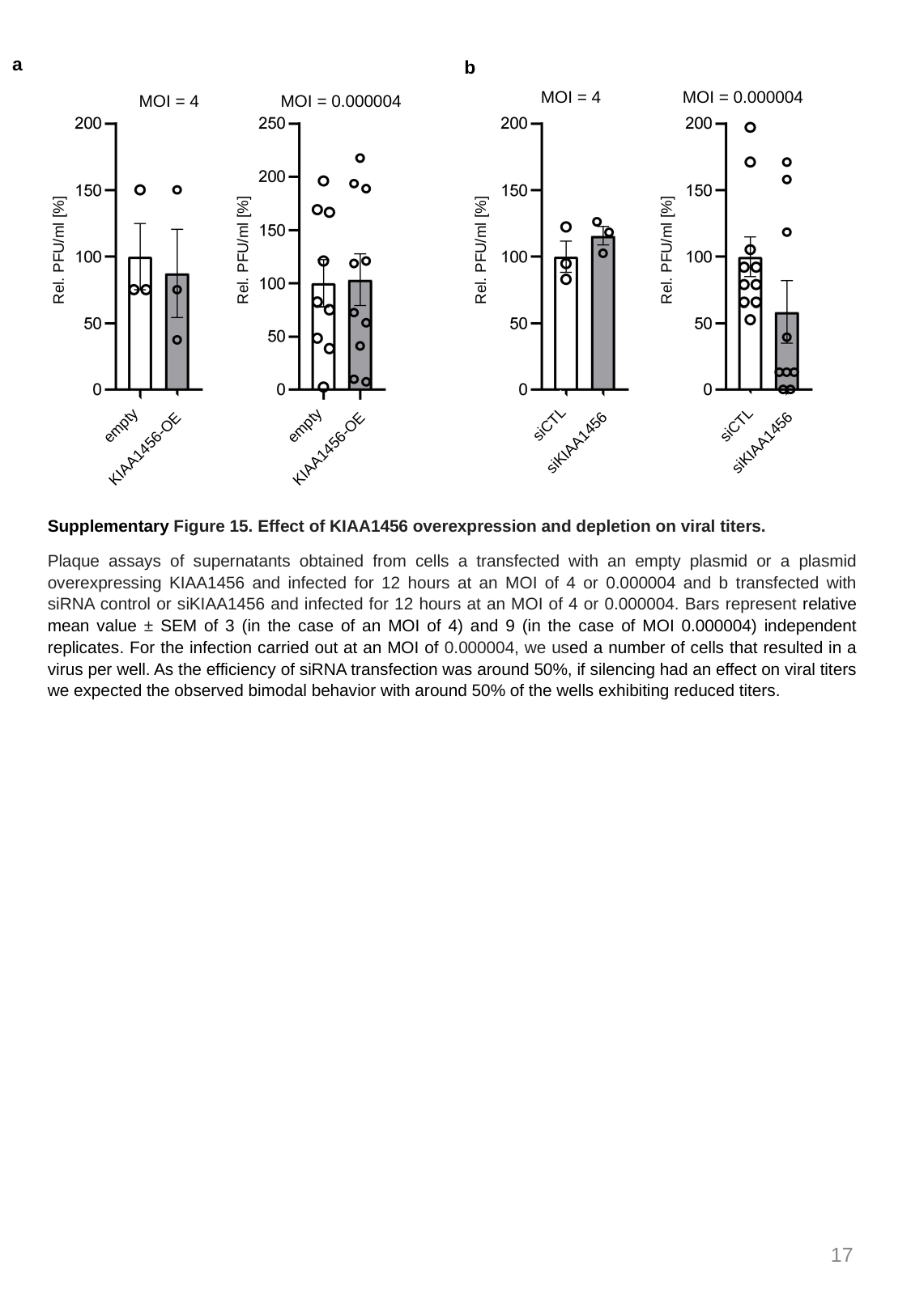

a
b
MOI = 4
MOI = 0.000004
MOI = 4
MOI = 0.000004
Rel. PFU/ml [%]
Rel. PFU/ml [%]
Rel. PFU/ml [%]
Rel. PFU/ml [%]
siCTL
siCTL
empty
empty
siKIAA1456
siKIAA1456
KIAA1456-OE
KIAA1456-OE
Supplementary Figure 15. Effect of KIAA1456 overexpression and depletion on viral titers.
Plaque assays of supernatants obtained from cells a transfected with an empty plasmid or a plasmid overexpressing KIAA1456 and infected for 12 hours at an MOI of 4 or 0.000004 and b transfected with siRNA control or siKIAA1456 and infected for 12 hours at an MOI of 4 or 0.000004. Bars represent relative mean value ± SEM of 3 (in the case of an MOI of 4) and 9 (in the case of MOI 0.000004) independent replicates. For the infection carried out at an MOI of 0.000004, we used a number of cells that resulted in a virus per well. As the efficiency of siRNA transfection was around 50%, if silencing had an effect on viral titers we expected the observed bimodal behavior with around 50% of the wells exhibiting reduced titers.
17

## Slide 18
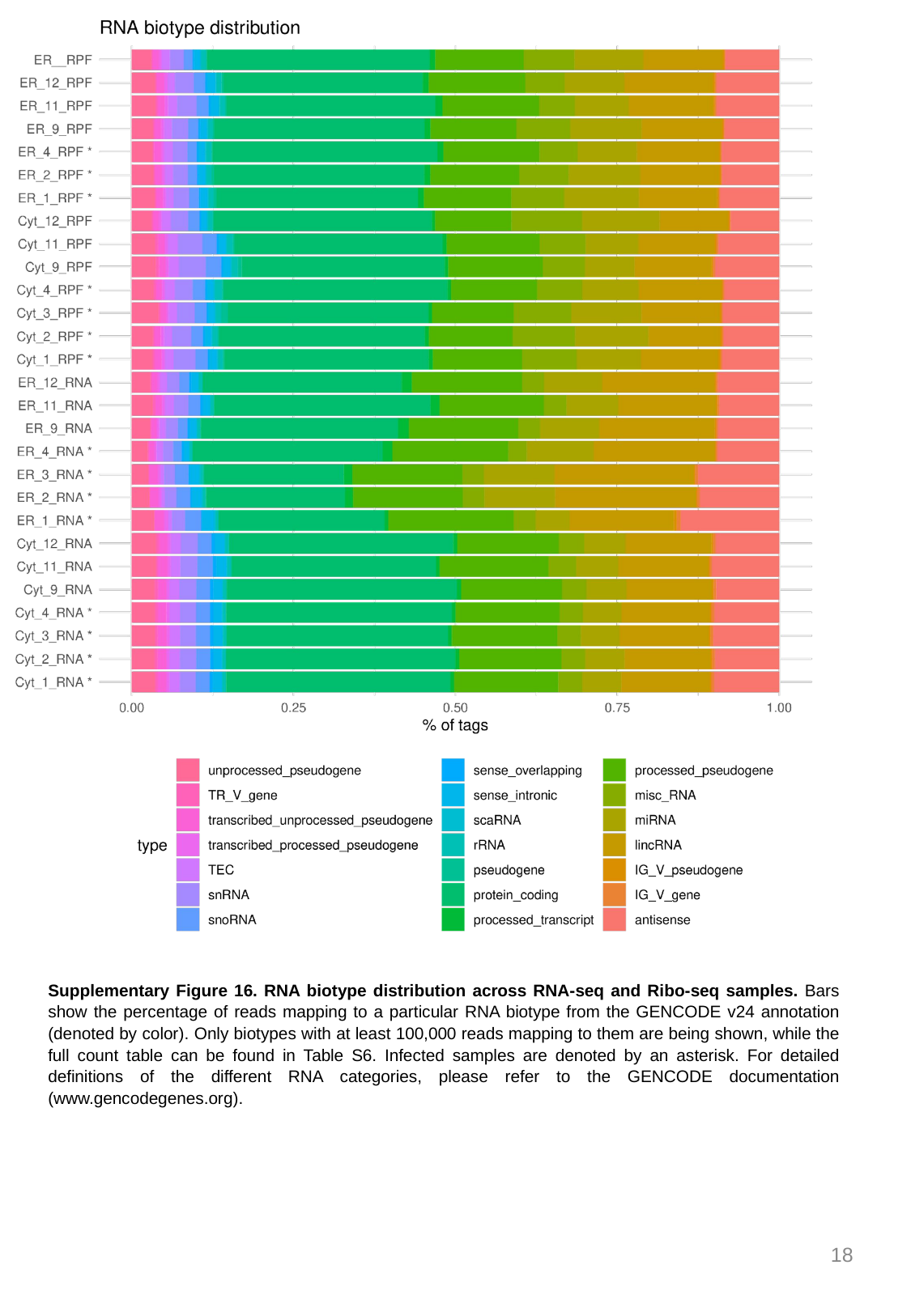

Supplementary Figure 16. RNA biotype distribution across RNA-seq and Ribo-seq samples. Bars show the percentage of reads mapping to a particular RNA biotype from the GENCODE v24 annotation (denoted by color). Only biotypes with at least 100,000 reads mapping to them are being shown, while the full count table can be found in Table S6. Infected samples are denoted by an asterisk. For detailed definitions of the different RNA categories, please refer to the GENCODE documentation (www.gencodegenes.org).
18

## Slide 19
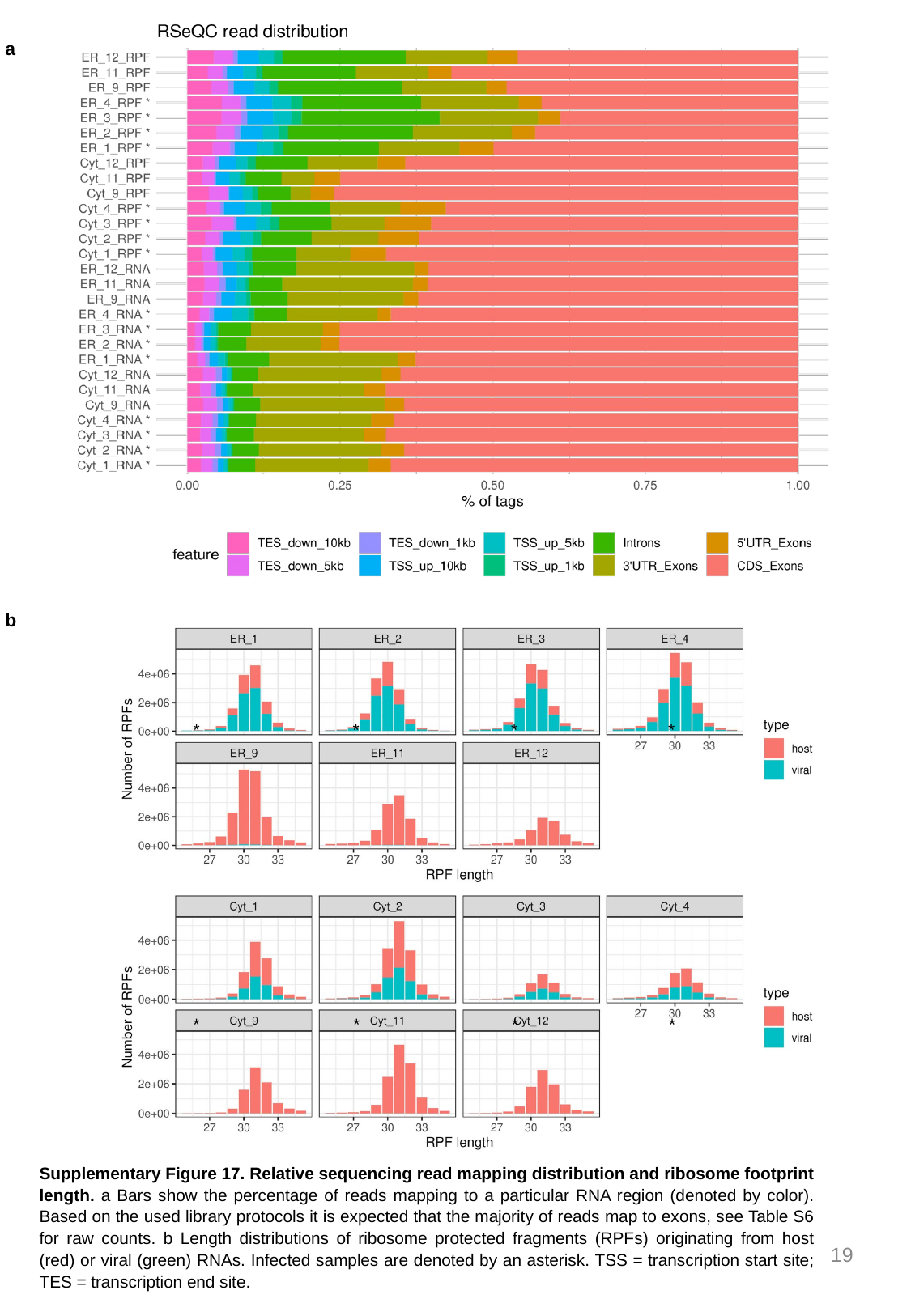

a
b
*
*
*
*
*
*
*
*
Supplementary Figure 17. Relative sequencing read mapping distribution and ribosome footprint length. a Bars show the percentage of reads mapping to a particular RNA region (denoted by color). Based on the used library protocols it is expected that the majority of reads map to exons, see Table S6 for raw counts. b Length distributions of ribosome protected fragments (RPFs) originating from host (red) or viral (green) RNAs. Infected samples are denoted by an asterisk. TSS = transcription start site; TES = transcription end site.
19

## Slide 20
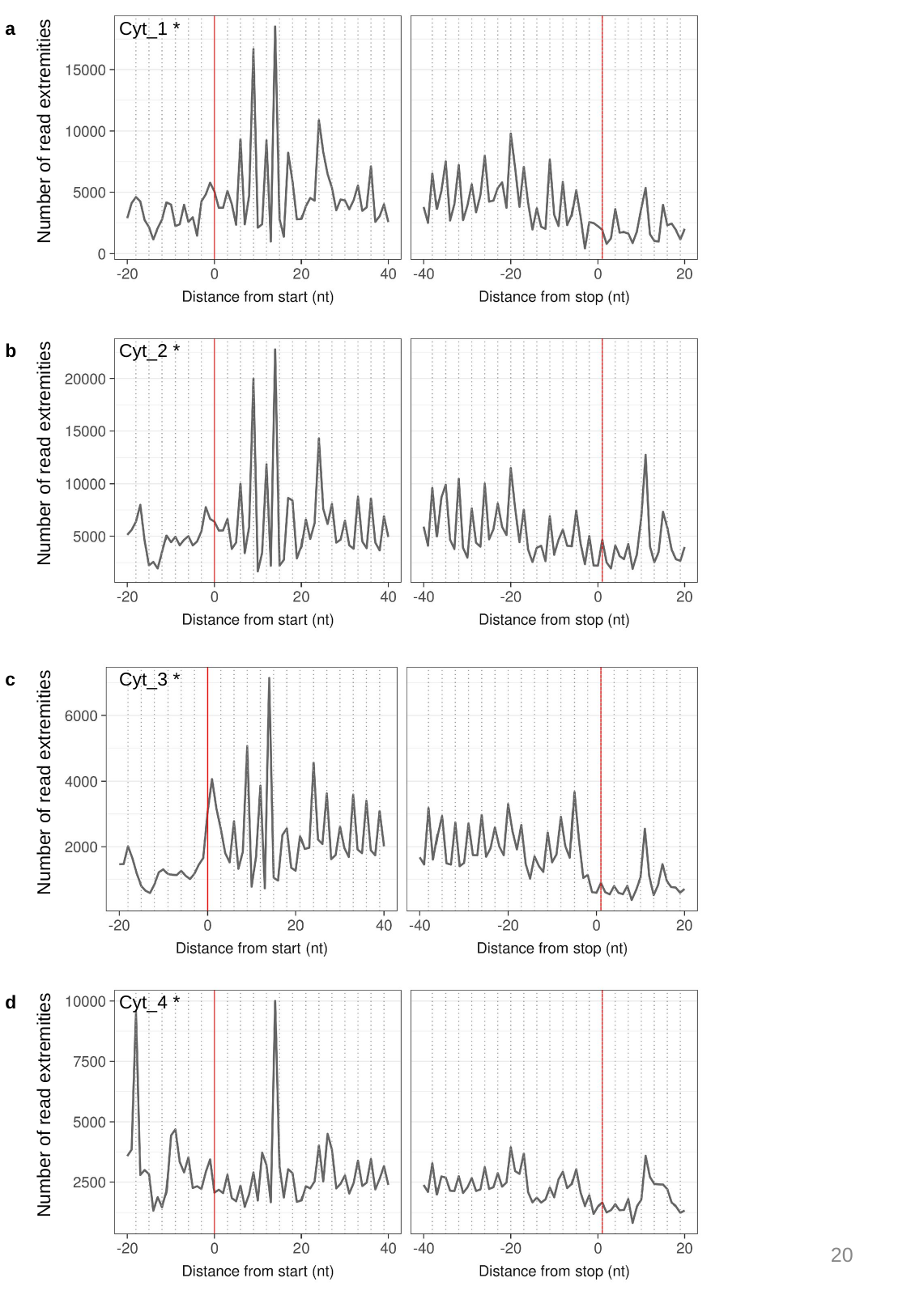

Cyt_1 *
Number of read extremities
a
Cyt_2 *
Number of read extremities
b
Cyt_3 *
Number of read extremities
c
Cyt_4 *
Number of read extremities
d
20

## Slide 21
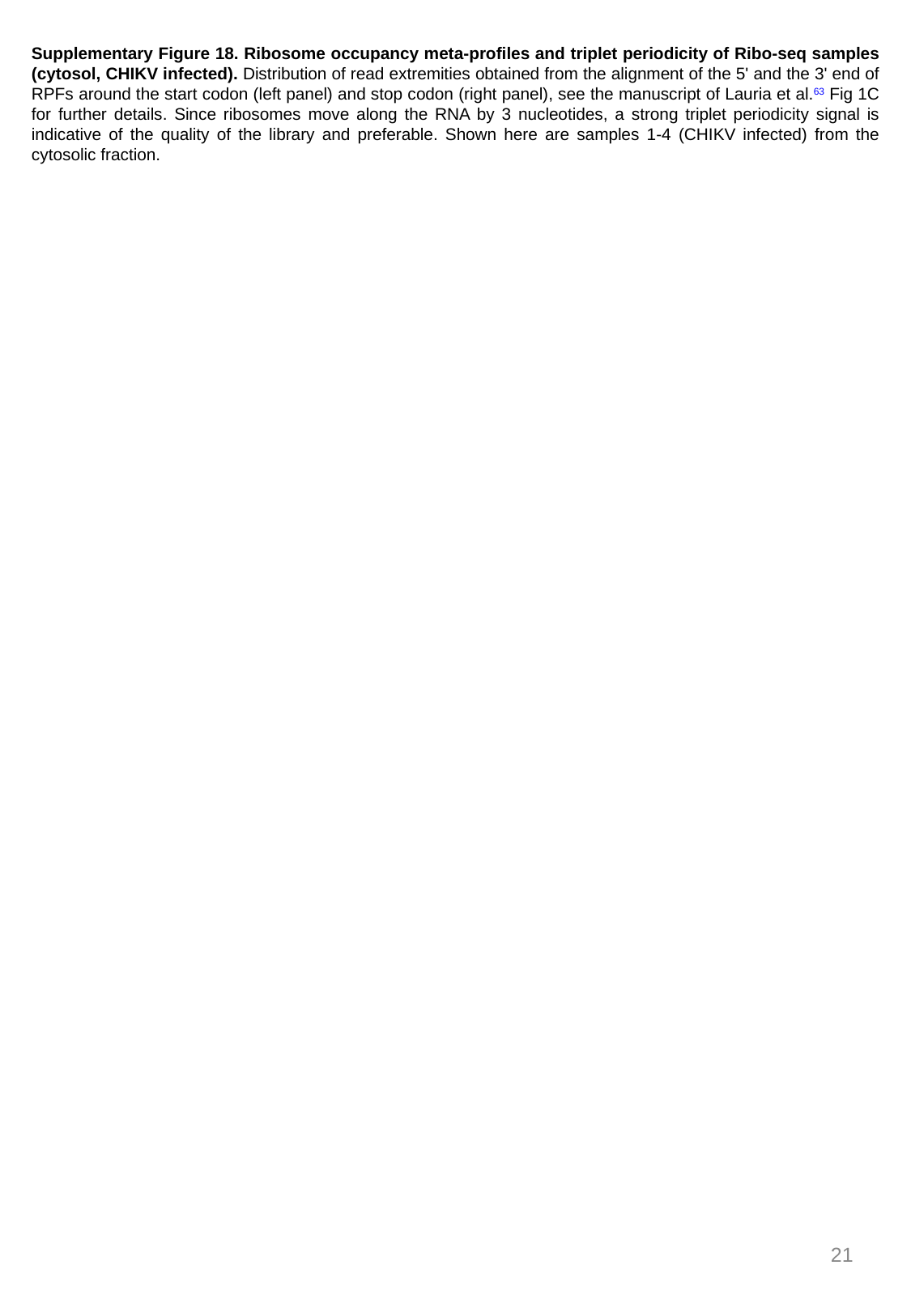

Supplementary Figure 18. Ribosome occupancy meta-profiles and triplet periodicity of Ribo-seq samples (cytosol, CHIKV infected). Distribution of read extremities obtained from the alignment of the 5' and the 3' end of RPFs around the start codon (left panel) and stop codon (right panel), see the manuscript of Lauria et al.63 Fig 1C for further details. Since ribosomes move along the RNA by 3 nucleotides, a strong triplet periodicity signal is indicative of the quality of the library and preferable. Shown here are samples 1-4 (CHIKV infected) from the cytosolic fraction.
21

## Slide 22
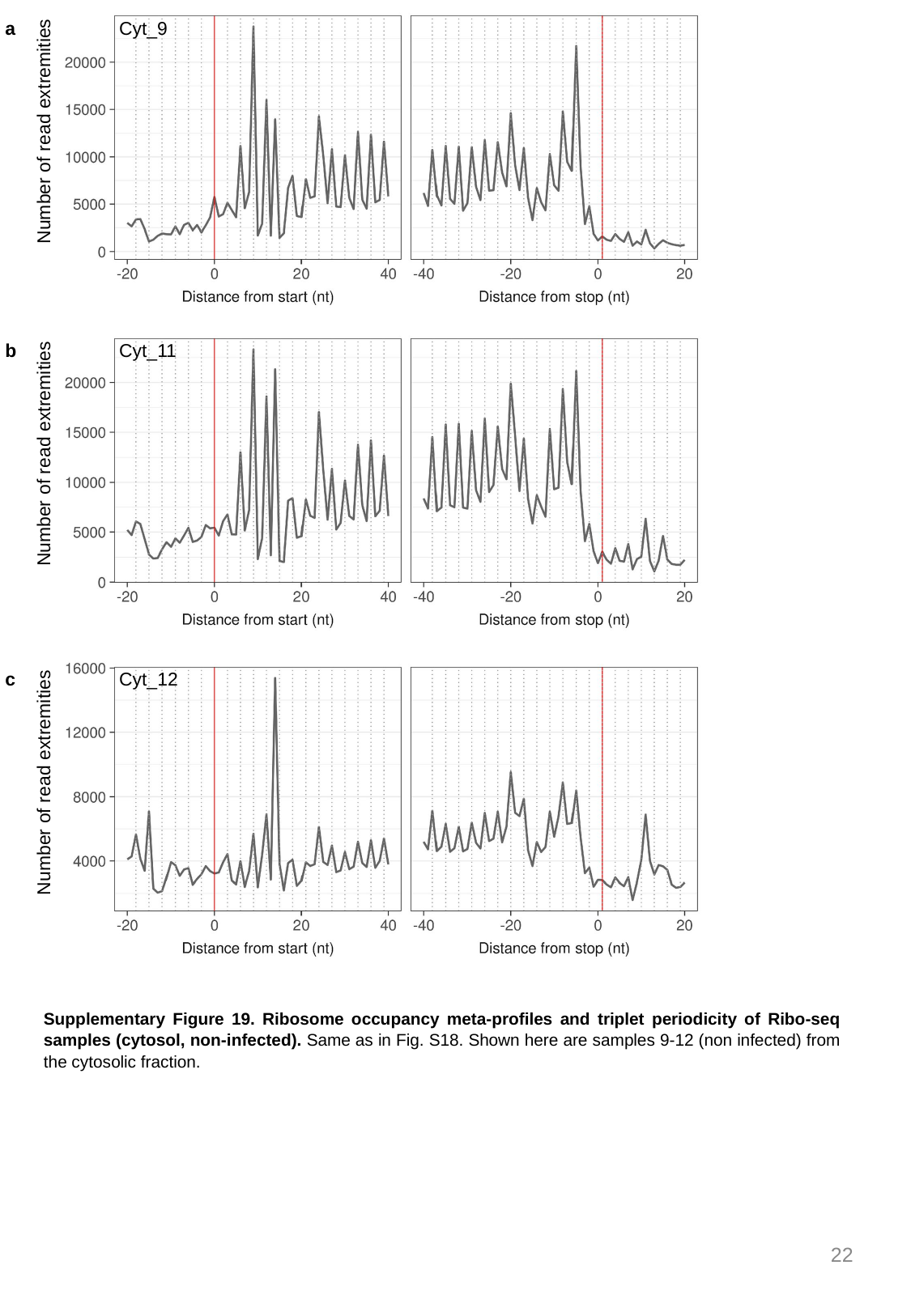

Cyt_9
Number of read extremities
a
Cyt_11
Number of read extremities
b
Cyt_12
Number of read extremities
c
Supplementary Figure 19. Ribosome occupancy meta-profiles and triplet periodicity of Ribo-seq samples (cytosol, non-infected). Same as in Fig. S18. Shown here are samples 9-12 (non infected) from the cytosolic fraction.
22

## Slide 23
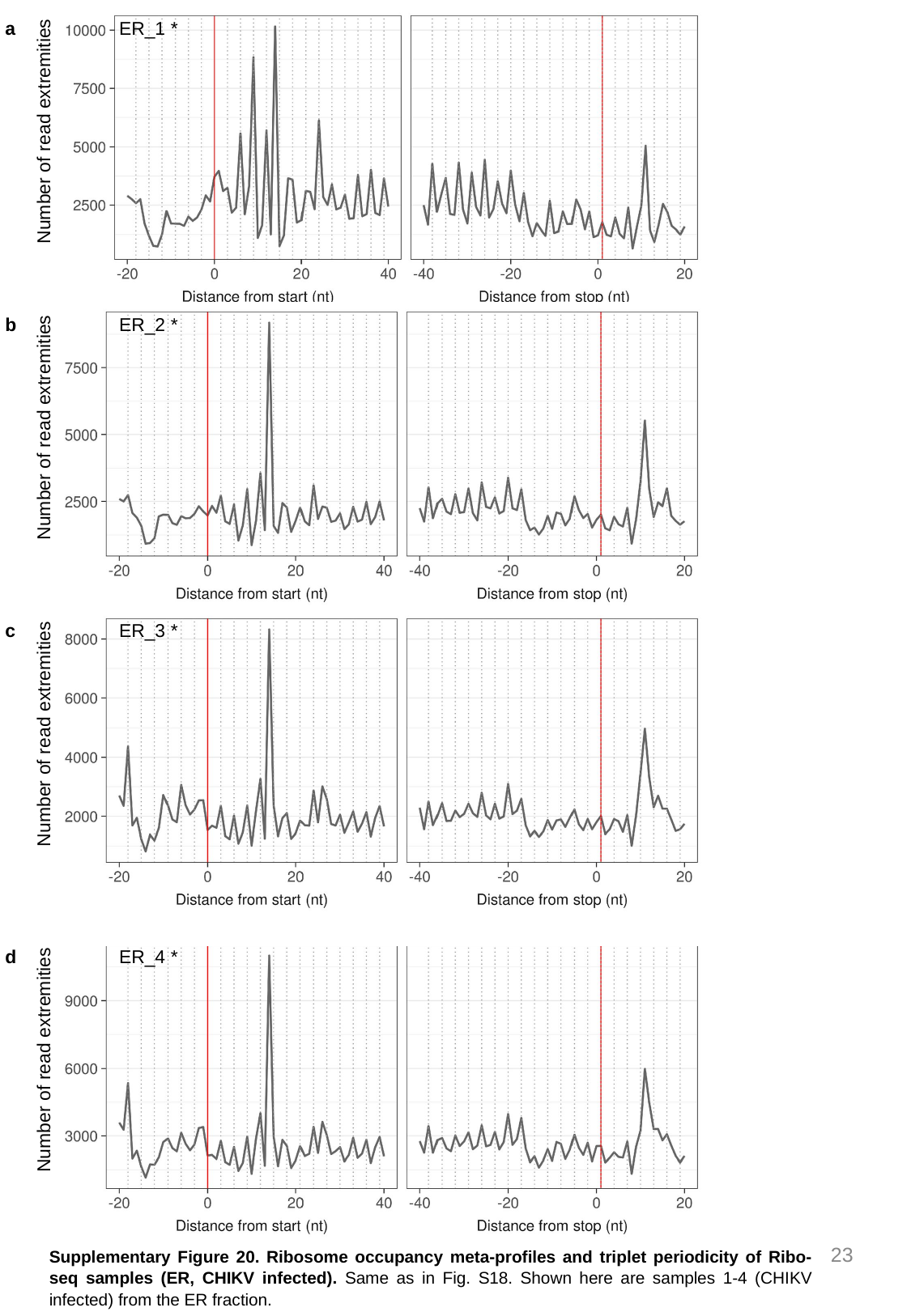

ER_1 *
Number of read extremities
a
ER_2 *
Number of read extremities
b
ER_3 *
Number of read extremities
c
ER_4 *
d
Number of read extremities
23
Supplementary Figure 20. Ribosome occupancy meta-profiles and triplet periodicity of Ribo-seq samples (ER, CHIKV infected). Same as in Fig. S18. Shown here are samples 1-4 (CHIKV infected) from the ER fraction.

## Slide 24
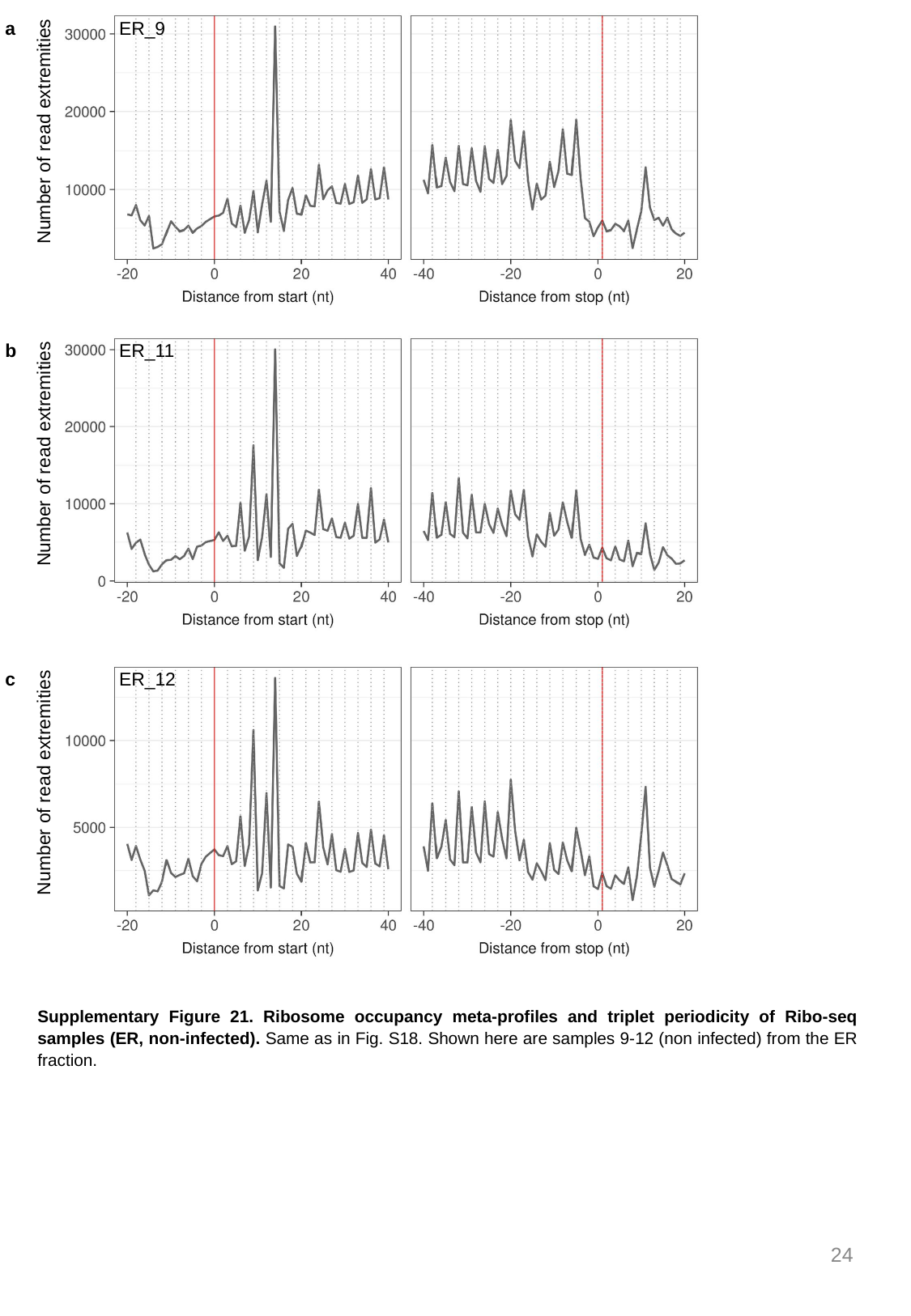

ER_9
Number of read extremities
a
ER_11
Number of read extremities
b
ER_12
Number of read extremities
c
Supplementary Figure 21. Ribosome occupancy meta-profiles and triplet periodicity of Ribo-seq samples (ER, non-infected). Same as in Fig. S18. Shown here are samples 9-12 (non infected) from the ER fraction.
24

## Slide 25
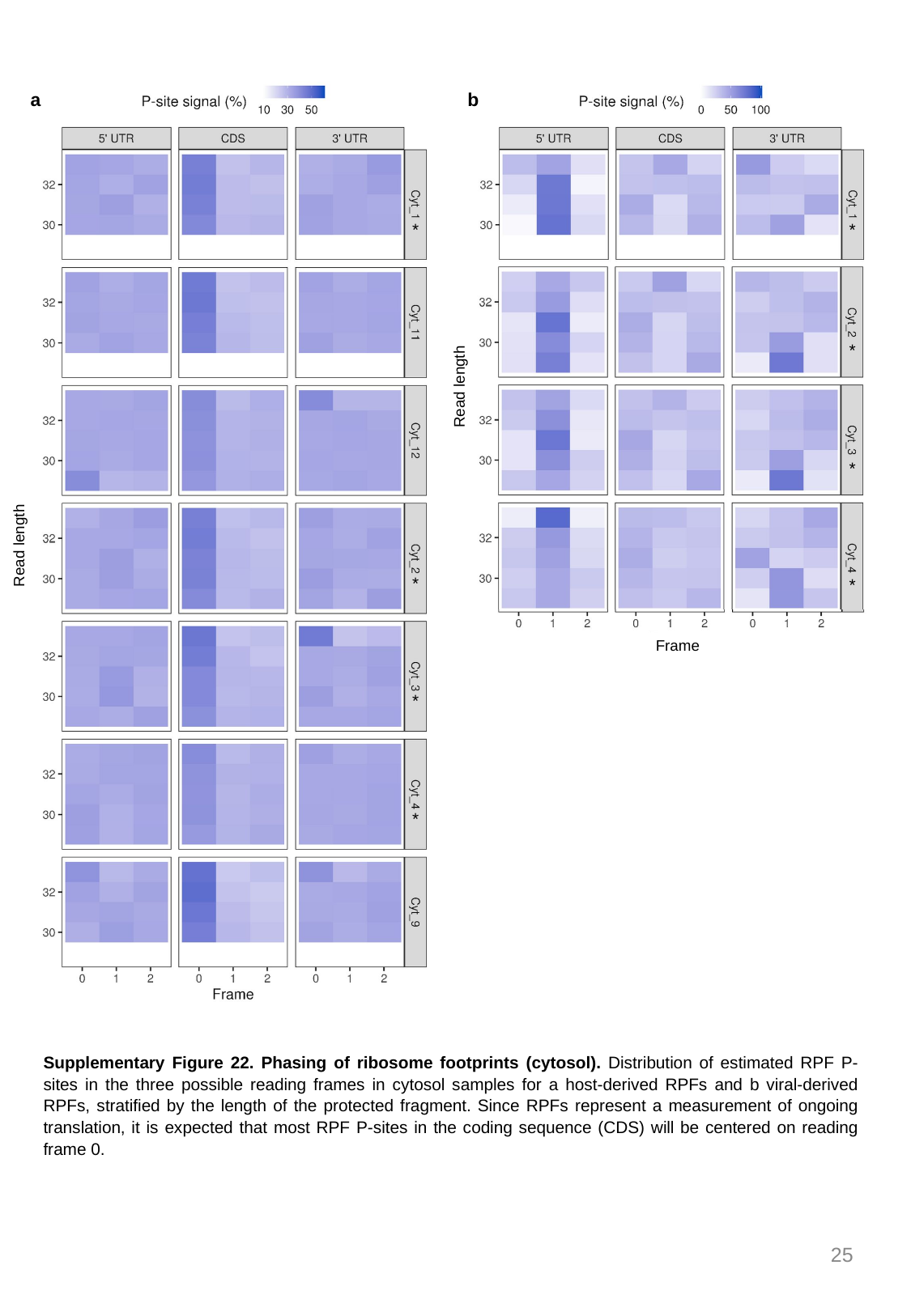

a
b
*
*
*
Read length
*
Read length
*
*
Frame
*
*
Supplementary Figure 22. Phasing of ribosome footprints (cytosol). Distribution of estimated RPF P-sites in the three possible reading frames in cytosol samples for a host-derived RPFs and b viral-derived RPFs, stratified by the length of the protected fragment. Since RPFs represent a measurement of ongoing translation, it is expected that most RPF P-sites in the coding sequence (CDS) will be centered on reading frame 0.
25

## Slide 26
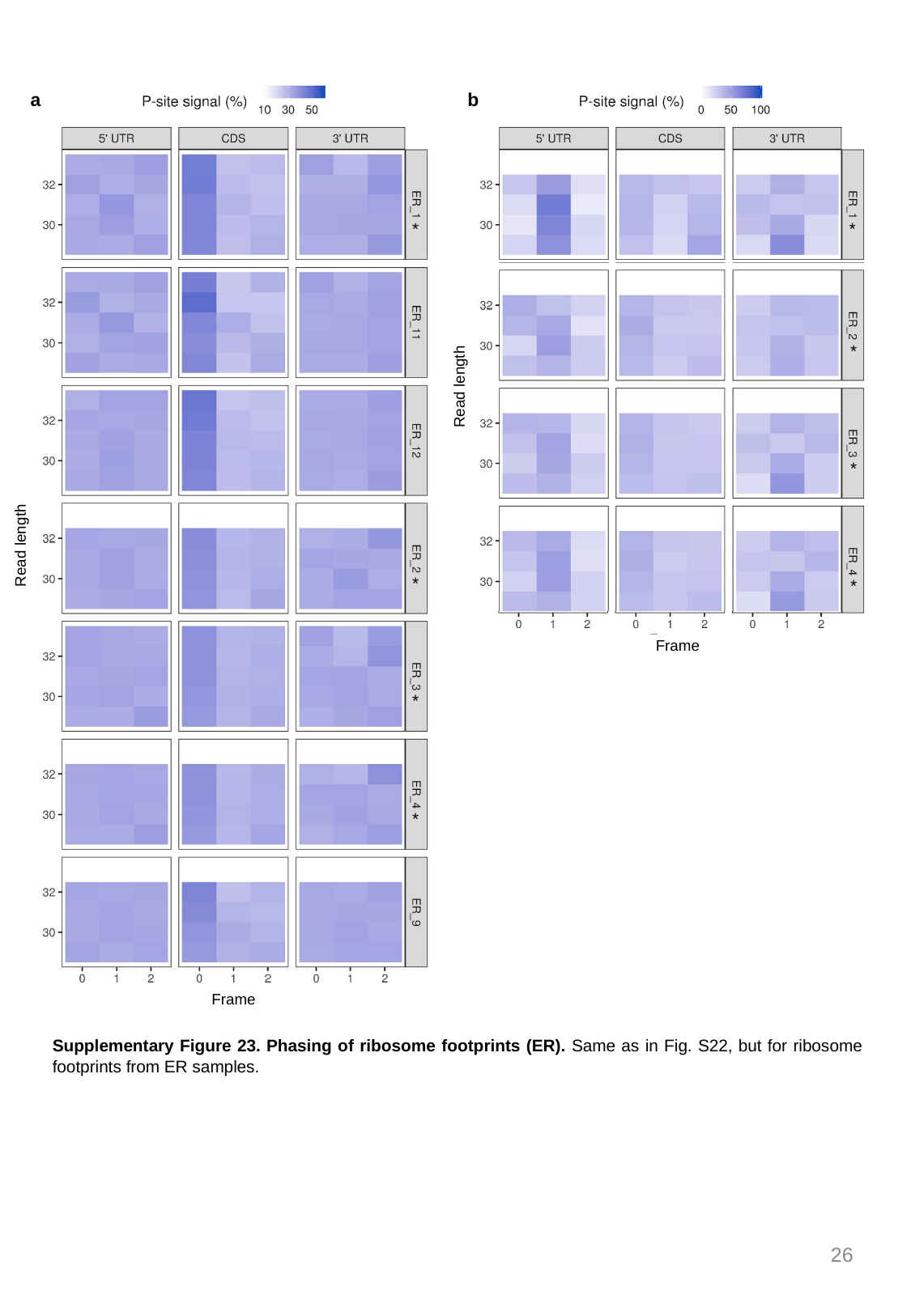

a
b
*
*
*
Read length
*
Read length
*
*
*
Frame
*
*
Frame
Supplementary Figure 23. Phasing of ribosome footprints (ER). Same as in Fig. S22, but for ribosome footprints from ER samples.
26

## Slide 27
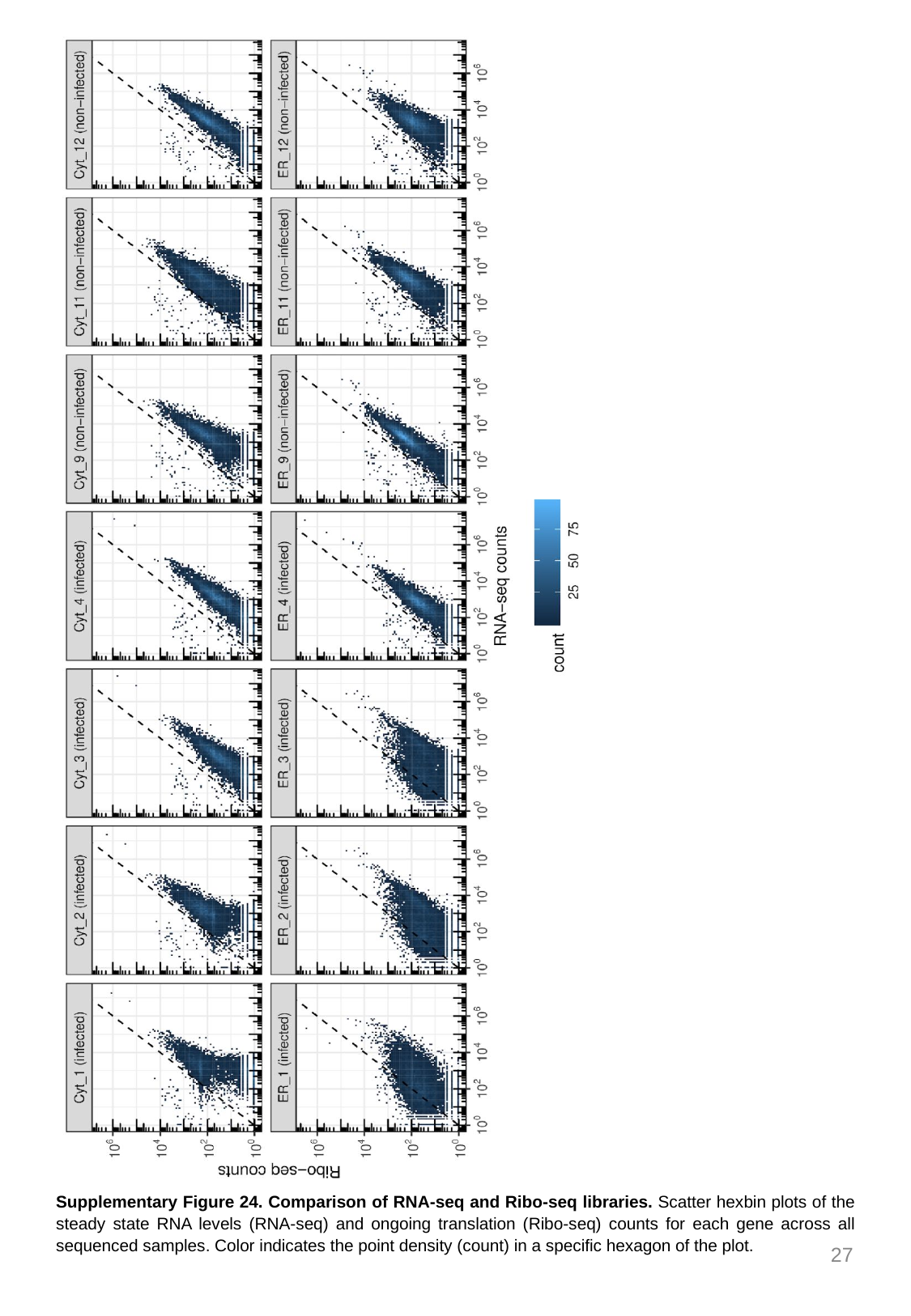

Supplementary Figure 24. Comparison of RNA-seq and Ribo-seq libraries. Scatter hexbin plots of the steady state RNA levels (RNA-seq) and ongoing translation (Ribo-seq) counts for each gene across all sequenced samples. Color indicates the point density (count) in a specific hexagon of the plot.
27

## Slide 28
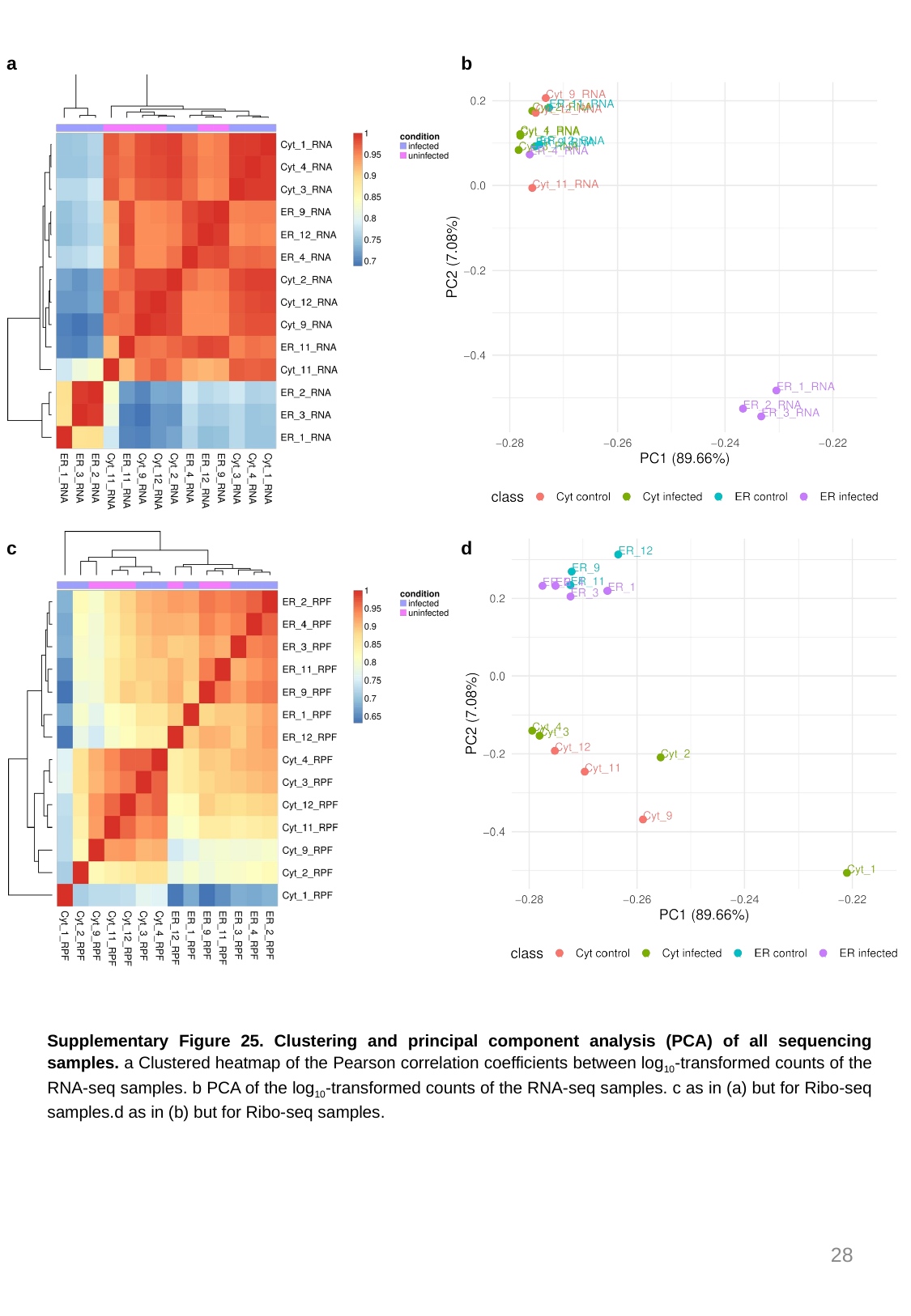

a
b
c
d
Supplementary Figure 25. Clustering and principal component analysis (PCA) of all sequencing samples. a Clustered heatmap of the Pearson correlation coefficients between log10-transformed counts of the RNA-seq samples. b PCA of the log10-transformed counts of the RNA-seq samples. c as in (a) but for Ribo-seq samples.d as in (b) but for Ribo-seq samples.
28

## Slide 29
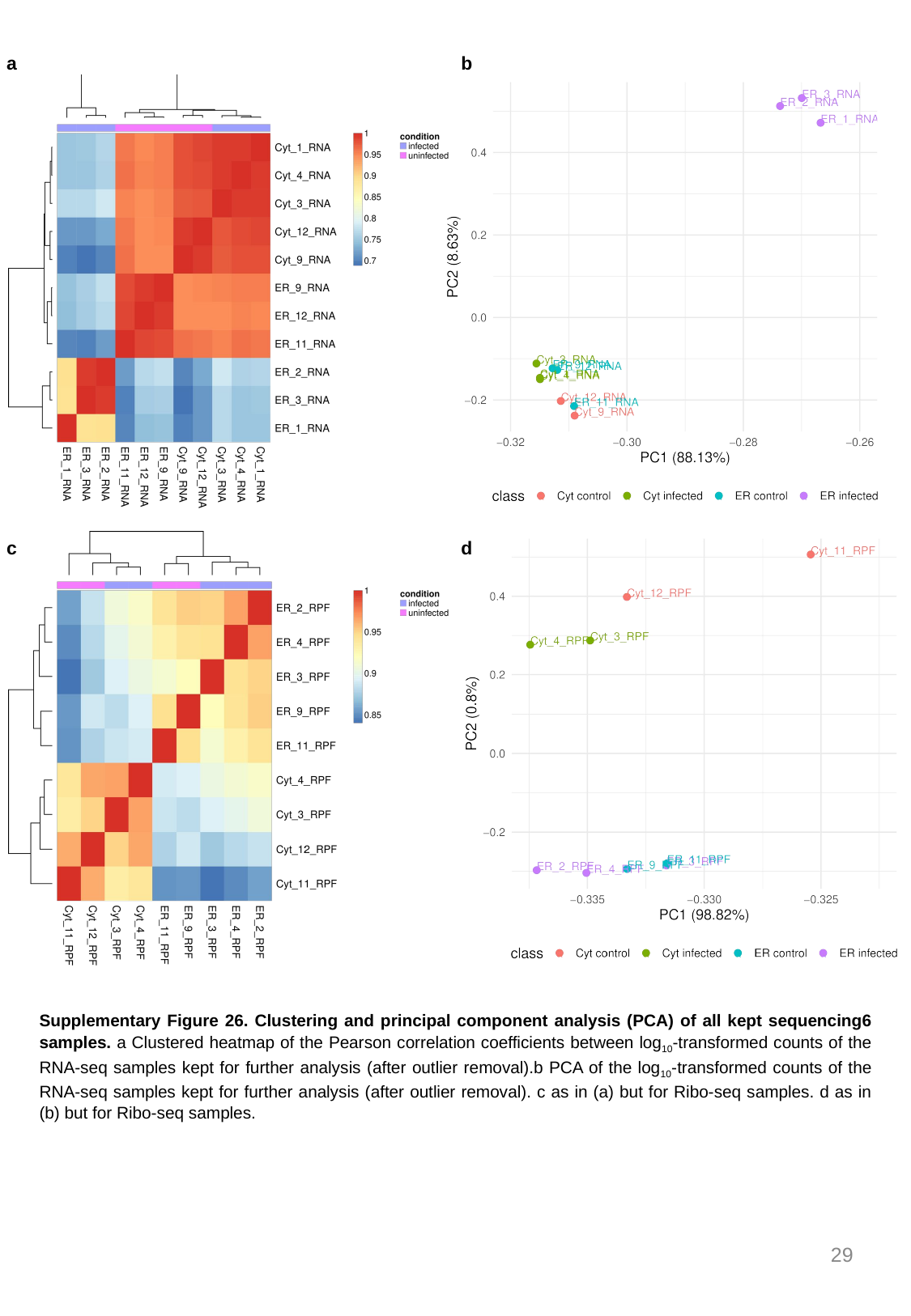

a
b
c
d
Supplementary Figure 26. Clustering and principal component analysis (PCA) of all kept sequencing6 samples. a Clustered heatmap of the Pearson correlation coefficients between log10-transformed counts of the RNA-seq samples kept for further analysis (after outlier removal).b PCA of the log10-transformed counts of the RNA-seq samples kept for further analysis (after outlier removal). c as in (a) but for Ribo-seq samples. d as in (b) but for Ribo-seq samples.
29

## Slide 30
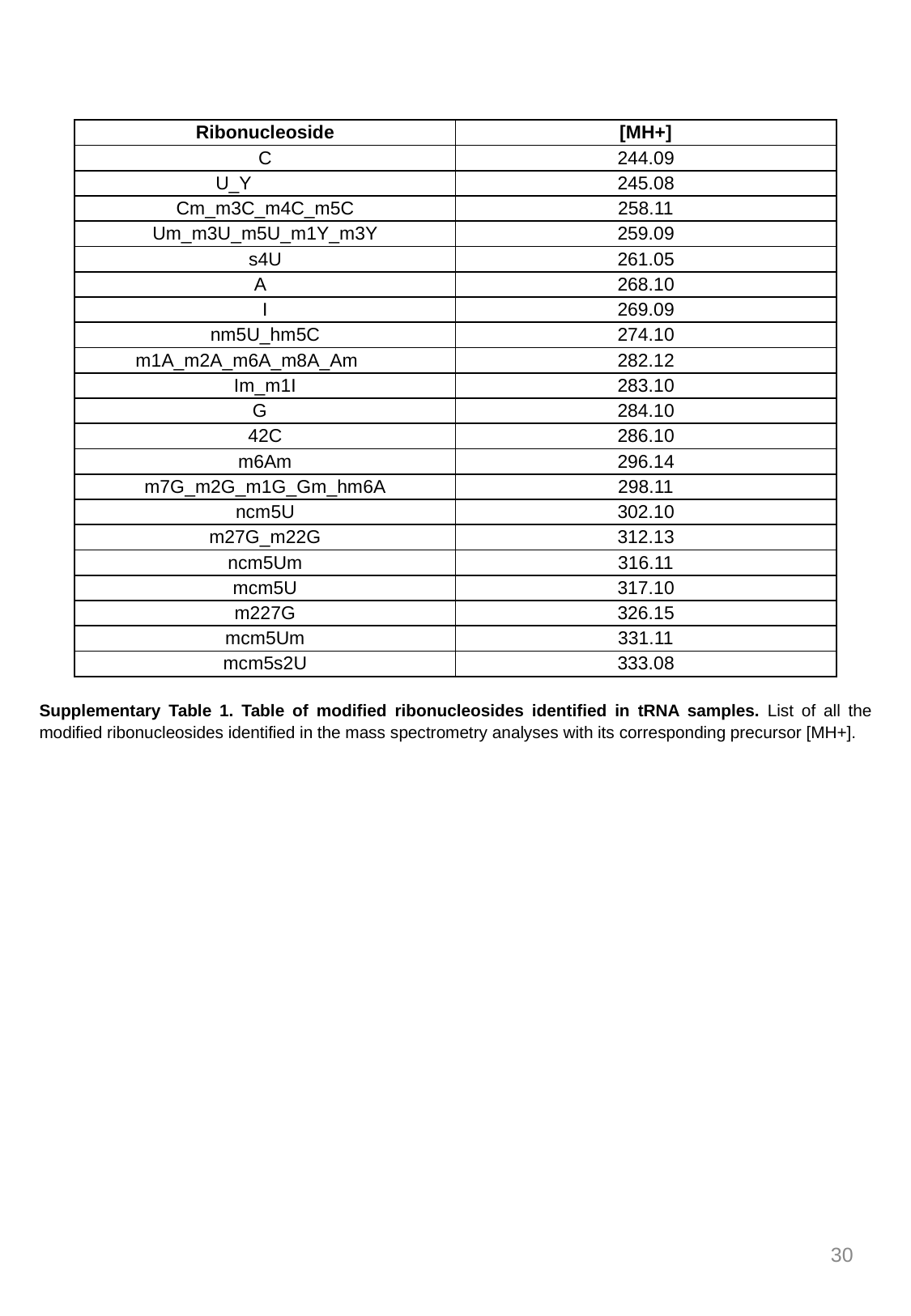

| Ribonucleoside | [MH+] |
| --- | --- |
| C | 244.09 |
| U\_Y | 245.08 |
| Cm\_m3C\_m4C\_m5C | 258.11 |
| Um\_m3U\_m5U\_m1Y\_m3Y | 259.09 |
| s4U | 261.05 |
| A | 268.10 |
| I | 269.09 |
| nm5U\_hm5C | 274.10 |
| m1A\_m2A\_m6A\_m8A\_Am | 282.12 |
| Im\_m1I | 283.10 |
| G | 284.10 |
| 42C | 286.10 |
| m6Am | 296.14 |
| m7G\_m2G\_m1G\_Gm\_hm6A | 298.11 |
| ncm5U | 302.10 |
| m27G\_m22G | 312.13 |
| ncm5Um | 316.11 |
| mcm5U | 317.10 |
| m227G | 326.15 |
| mcm5Um | 331.11 |
| mcm5s2U | 333.08 |
Supplementary Table 1. Table of modified ribonucleosides identified in tRNA samples. List of all the modified ribonucleosides identified in the mass spectrometry analyses with its corresponding precursor [MH+].
30
