# Supplementary material for: CHIKV infection reprograms codon optimality to favor viral RNA translation by altering the tRNA epitranscriptome
Source: Nat Commun. 2022 Aug 11;13:4725. doi: 10.1038/s41467-022-31835-x (PMC9366759; doi:10.1038/s41467-022-31835-x)
Supplement: Supplementary file 3 — Description of additional Supplementary Data [file 41467_2022_31835_MOESM3_ESM.docx]

**CHIKV infection reprograms codon optimality to favor viral RNA translation by altering the tRNA epitranscriptome**

Jungfleisch *et al*.

**Description of Additional supplementary Data**

**Title: Supplementary Data 1**

**Description: Comprehensive overview of differential ribosome occupancy results.** Sheet “RNA_only”: results of the differential expression (DE) analysis obtained by analyzing ER and cytosol using limma-voom, respectively. Columns contain: gene – ensembl gene ID; gene symbol – official gene symbol; class – the behavior of the mRNA in both compartments relative to non-infected condition; summarized_class – the assigned class as depicted in Figure 1b; Cyt_log_2_FC_RNA / Cyt_FDR_RNA – limma-voom log_2_ fold change and Benjamini-Hochberg adjusted p-value (FDR) of the RNA in the cytosol (infected vs. non-infected); ER log_2_FC_RNA; FDR_RNA_ER – same as Cyt_log_2_FC_RNA and Cyt_FDR_RNA, but for ER translation_class_Cyt – whether translation of the RNA was activated, repressed or not significantly altered in the cytosol upon CHIKV infection (see Fig. 2c); translation class ER - same as translation class cytosol but for ER (see Fig. 2d). Sheets “ER” and “Cyt”: results of the differential translation analysis obtained by analyzing ER and cytosol using limma-voom, respectively. Columns contain: gene / transcript – ensembl gene / transcript ID; gene symbol – official gene symbol; FDR_translation – Benjamini-Hochberg adjusted p-value (FDR) of the limma-voom analysis using an interaction term to identify differential ribosome occupancy; class – class assigned based on differential translation analysis taking into account RNA and RPF changes; log_2_FC_RNA / FDR_RNA – limma-voom log_2_ fold change and FDR when only testing for changes in RNA abundance after CHIKV infection in the respective compartment, see sheet “RNA_only”; log_2_FC_RPF / FDR_RPF – limma-voom log_2_ fold change and FDR when only testing for changes in RPF levels after CHIKV infection. nRF_non-infected / rRF_infected –normalized ribosome footprints in each condition; nRF_ratio – ratio of nRF_infected / nRF_non-infected; mean_CPM_RNA_non-infected / mean_CPM_RNA_infected / mean_CPM_RPF_non-infected / mean_CPM_RPF_infected – mean counts per million (CPM) of RNA-seq reads (RNA) or Ribo-seq reads (RPF) in the respective condition.

**Title: Supplementary Data 2**

**Description: GO terms for different classes of RNAs depicted in Figure 1b.** Individual classes are stored in separate sheets. GO enrichments were calculated using the hypergeometric test and gSCS multiple testing correction as implemented in the gProfileR R package. Columns represent: domain – the queried database (GO = gene ontology terms, KEGG = KEGG pathways, REACTOME = REACTOME pathways). For GO, BP = biological process, CC = cellular component and MF = molecular function; p.value – adjusted p-value from gProfileR; term.name – description of the enriched term; term.id – systematic ID of the term; term.size – number of genes associated to term in database; query.size – number of query genes recognized by database; overlap.size – number of recognized query genes associated to term; intersection – identifiers of recognized query genes associated to term.

**Title: Supplementary Data 3**

**Description: GO terms for translationally activated and repressed genes at the ER.** GO enrichments were calculated using the hypergeometric test and gSCS multiple testing correction as implemented in the gProfileR R package. Columns as in Supplementary Table 2.

**Title: Supplementary Data 4**

**Description: Translational behavior of known RNA modification enzymes.** Sheets “ER” and “Cyt”: results of differential ribosome occupancy obtained by analyzing ER and cytosol using limma-voom, respectively. Column names correspond to Supplementary Table 1. Additional columns: modification – modification(s) mediated by the RNA modification enzyme; position – position(s) in the tRNA affected by modification. Italicized entries denote modifications and positions with lower confidence.

**Title: Supplementary Data 5**

**Description: Percentage of GAA, AAA, CAA, AGA and GGA codons in multiple viral (+)RNA genomes.** Columns contain: Species – whether human or features of a particular virus are shown; Assembly: the corresponding assembly used; CAI – the codon adaptation index for a viral genome or group of RNAs (indicated by *); # codons total – the total number of codons in the viral genome or group of RNAs; # A-ending / # C-ending / # G-ending / # T-ending - the number of A, C, G and T-ending codons in the viral genome or group of RNAs; % A / % C / % G / % T – the percentage of A, C, G and T-ending codons; # GAA, AAA, CAA, AGA and GGA codons / % GAA, AAA, CAA, AGA and GGA codons – the number and percentage of GAA, AAA, CAA, AGA and GGA codons found in the viral genome or group of RNAs.

**Title: Supplementary Data 6**

**Description: Raw counts used to generate the RNA biotype distribution (Fig. S16) and sequencing read mapping distribution (Fig. S17) plots.** The two tables are stored in individual sheets and contain all biotypes and regions included in the analyses.

**Title: Supplementary Data 7**

**Description: Overview of sequencing samples (RNA-seq and Ribo-seq).** Columns contain: sample – the sample name; condition – whether the sample was an uninfected control or infected with CHIKV; excluded – whether any one or both of the sequencing libraries for a particular sample were excluded from the analysis after QC; RNA-seq reads – the total number of RNA-seq reads for this library; Mapped reads – host – the number of RNA-seq reads mapping to host genes; Mapped reads – CHIKV – the number of reads mapping to the CHIKV genome; Mapped reads – total – the sum of the previous two columns; Ribo-seq reads – the total number of Ribo-seq reads for this library; Mapped RPF – host – the number of Ribo-seq reads (RPF) mapping to host genes; Mapped RPF – CHIKV – the number of Ribo-seq reads (RPF) mapping to the CHIKV genome; Mapped RPF – total – the sum of the previous two columns.
