# Supplementary material for: CHIKV infection reprograms codon optimality to favor viral RNA translation by altering the tRNA epitranscriptome
Source: Nat Commun. 2022 Aug 11;13:4725. doi: 10.1038/s41467-022-31835-x (PMC9366759; doi:10.1038/s41467-022-31835-x)
Supplement: Supplementary file 11 — Reporting Summary [file 41467_2022_31835_MOESM11_ESM.pdf]

nature research

Corresponding author(s): Juana Díez, René Böttcher

Last updated by author(s): Jun 17, 2022

Reporting Summary

Nature Research wishes to improve the reproducibility of the work that we publish. This form provides structure for consistency and transparency in reporting. For further information on Nature Research policies, see our Editorial Policies and the Editorial Policy Checklist.

Statistics

For all statistical analyses, confirm that the following items are present in the figure legend, table legend, main text, or Methods section.

n/a	Confirmed	
☐	☒	The exact sample size (n) for each experimental group/condition, given as a discrete number and unit of measurement
☐	☒	A statement on whether measurements were taken from distinct samples or whether the same sample was measured repeatedly
☐	☒	The statistical test(s) used AND whether they are one- or two-sided<br>*Only common tests should be described solely by name; describe more complex techniques in the Methods section.*
☒	☐	A description of all covariates tested
☐	☒	A description of any assumptions or corrections, such as tests of normality and adjustment for multiple comparisons
☐	☒	A full description of the statistical parameters including central tendency (e.g. means) or other basic estimates (e.g. regression coefficient) AND variation (e.g. standard deviation) or associated estimates of uncertainty (e.g. confidence intervals)
☐	☒	For null hypothesis testing, the test statistic (e.g. F, t, r) with confidence intervals, effect sizes, degrees of freedom and P value noted<br>*Give P values as exact values whenever suitable.*
☒	☐	For Bayesian analysis, information on the choice of priors and Markov chain Monte Carlo settings
☒	☐	For hierarchical and complex designs, identification of the appropriate level for tests and full reporting of outcomes
☐	☒	Estimates of effect sizes (e.g. Cohen's d, Pearson's r), indicating how they were calculated

Our web collection on statistics for biologists contains articles on many of the points above.

Software and code

Policy information about availability of computer code

Data collection	No custom software was used.
Data analysis	Custom transcriptome generation based on expressed gene isoforms: kallisto v0.43.0; sleuth v0.29.0; R v3.4.<br>Preprocessing of Ribo-seq reads: cutadapt v1.91, settings: -a CTGTAGGCACCATCAAT --quality-base 33 -q 28,28 -m 25 -M 35<br>Mapping of Ribo-seq reads to custom transcriptome: bowtie v1.2.1, default settings.<br>Downstream statistical analyses, p-site assignment: R 3.4, riboWaltz v1.1.1<br>RNA-seq read mapping: TopHat2 v2.1.0, settings: --max-multihits 1 --b2-very-sensitive --no-coverage-search --library-type fr-firststrand<br>Read counting: featureCounts v1.5.1, settings: -s 2 -Q 1 -p<br>Differential expression / translation analysis: R v3.4; limma-voom v3.32.2<br>General visualizations: ggplot2 v2.2.1<br>Heatmaps: NMF v0.21.0 (aheatmap function)<br>GO term enrichment: gProfileR v0.6.4<br>CAI calculation: seqinR v3.4-5<br>Mass spectrometry analysis: Skyline v20.2.0.343<br>Imagegauge Software FIJI_ImageJ win64<br>Odysseey Software ImageStudio Lite Ver 5.2

For manuscripts utilizing custom algorithms or software that are central to the research but not yet described in published literature, software must be made available to editors and reviewers. We strongly encourage code deposition in a community repository (e.g. GitHub). See the Nature Research guidelines for submitting code & software for further information.

Data

Policy information about availability of data

All manuscripts must include a data availability statement. This statement should provide the following information, where applicable:

- Accession codes, unique identifiers, or web links for publicly available datasets
- A list of figures that have associated raw data
- A description of any restrictions on data availability

Data supporting the findings of this study are available within the article and its Supplementary Figures. The source data are provided as Source Data file. The Ribo-Seq and RNA-seq data from this study have been submitted to the NCBI Gene Expression Omnibus (GEO; http://www.ncbi.nlm.nih.gov/geo/) under accession number GSE143390. The raw proteomics data have been deposited to the MetabolLights study (www.ebi.ac.uk/metabolights/MTBLS2443)53 repository with the dataset identifier MTBLS2443.

Relevant data resources:

hg38 genome FASTA

https://hgdownload.soe.ucsc.edu/goldenPath/hg38/bigZips/hg38.fa.gz

CHIKV genome FASTA & annotation

https://www.ncbi.nlm.nih.gov/nuccore/KT449801.1

codon usage tables for different species

https://dnahive.fda.gov/dna.cgi?cmd=codon_usage&id=537&mode=cocoputs

Appris principal isoforms

https://appris.bioinfo.cnio.es/#/downloads

Field-specific reporting

Please select the one below that is the best fit for your research. If you are not sure, read the appropriate sections before making your selection.

☒ Life sciences ☐ Behavioural & social sciences ☐ Ecological, evolutionary & environmental sciences

For a reference copy of the document with all sections, see nature.com/documents/nr-reporting-summary-flat.pdf

Life sciences study design

All studies must disclose on these points even when the disclosure is negative.

Sample size	In general, no calculations were done to determine sample size. Sample size was determined based on standards for experimental studies attempting to have a minimum of N=3 biological replicates with sufficient reproducibility.
Data exclusions	Clear outliers were removed. Therefore, replicate quality was assessed by sample clustering and by performing a principal component analysis on the log10-transformed count matrix. Supplementary figures 25 and 26 contain a summary of the individual samples and whether they were excluded or not.
Replication	The results were either confirmed by performing the experiment multiple times with successful replication (at least in three independent replicates) or by complementarity approaches.
Randomization	Samples were allocated randomly for culture, infection, transfection and analysis. Data were then grouped according to their infection/transfection status.
Blinding	Blinding was not performed in our study. The experimental parameters determined in this study are considered as objective measures, no subject to bias and therefore the integrity of the results are not impacted when running the study and analysis unblinded.

Reporting for specific materials, systems and methods

We require information from authors about some types of materials, experimental systems and methods used in many studies. Here, indicate whether each material, system or method listed is relevant to your study. If you are not sure if a list item applies to your research, read the appropriate section before selecting a response.

Materials & experimental systems

n/a	Involved in the study
[ ]	[x] Antibodies
[ ]	[x] Eukaryotic cell lines
[x]	[ ] Palaeontology and archaeology
[x]	[ ] Animals and other organisms
[x]	[ ] Human research participants
[x]	[ ] Clinical data
[x]	[ ] Dual use research of concern

Methods

n/a	Involved in the study
[x]	[ ] ChIP-seq
[x]	[ ] Flow cytometry
[x]	[ ] MRI-based neuroimaging

Antibodies

Antibodies used

KIAA1456: ThermoFisher, PA5-70320 and PA5-77162
Grp94: Cell Signaling Technology, Cat#2104S
TRAP: Santa Cruz, Cat#sc-376875
Tubulin: Sigma-Aldrich, Cat#T9026, RRID: AB_477593
Vinculin: Sigma-Aldrich, Cat#V9131
SLC25A45: Novus Biological, Cat #NBP2-30521
PRSS16: Novus Biological, Cat #NBP1-91559
DRP2: Novus Biological, Cat #NBP2-16229
PHF7: ThermoFisher, Cat#TA505115
KSR2: Abcam, Cat#ab72753
CHIKV Nsp1 and Capsid: kindly provided by A. Merits (not commercially available, produced by A.Merits lab)
Fluorescence-conjugated secondary antibodies Li-cor IRDye 680RD donkey anti-rabbit Cat#926-68073 Lot#D00421-09 and Li-cor IRDye 800CW donkey anti-mouse Cat#926-32210.

Validation

KIAA1456 antibodies were validated in this study via overexpression and knockdown for the detection of endogenous levels of human KIAA1546 protein by WB (see Figure 5b, e,f and i)
Grp94 antibody was validated by Cell Signaling Technology in WB. Detects endogenous levels of total Grp94 protein. Species Reactivity: Human, mouse,rat and monkey. Application WB.
TRAP antibody was validated by Santa Cruz via WB and Immunofluorescence. Detects endogenous levels of total TRAP protein. Species Reactivity: Human. Application WB, IP, IF, IHC, ICC, ELISA.
Tubulin antibody was validated by Sigma Aldrich via WB and immunofluorescence. Detects endogenous levels of total tubulin protein. Species Reactivity: Human, mouse, drosophila and rat. Application WB, IF, ICC-IF and ICC.
Vinculin antibody was validated by Sigma Aldrich via WB. Detects endogenous levels of total vinculin protein. Species Reactivity: Human, mouse, rat and bos taurus. Application WB, IF, ICC-IF and ICC.
SLC25A45, PRSS16 and DRP2 antibodies were validated by Novus Biologicals via WB. SLC25A45 species Reactivity: Human. Application WB, IHC, ICC-IF and IHC-P. PRSS16 species reactivity: human. Application: WB. DRP2 species reactivity:
PHF7 antibody was validated by ThermoFisher in WB by overexpressing it. Species reactivity: Human. Applications: WB, IHC, ICC, Flow.
KSR2 was validated by abcam via WB. Species reactivity: Human. Applications: WB, ICC, IF.
CHIKV Nsp1 and Capsid protein antibody were validated in previous studies of A. Merits (Scholte et al., Journal of Virology, 2015, Vol89, No.8; https://doi.org/10.1128/JVI.03612-14 & Taylor et al., ASM Journals, 2017, Vol.8, No.1; https://doi.org/10.1128/mBio.01970-16)

Eukaryotic cell lines

Policy information about cell lines

Cell line source(s)

Human embryonic kidney cells (Hek293T): ATCC; CRL-11268; https://www.atcc.org/products/crl-11268
Huh7/Scr: kindly provided by Francis Chisari (originally obtained from ThermoFisherScientific).
Fibroblasts BHK-21 [C31]: ATCC; CCL-10; https://www.atcc.org/products/ccl-10

Authentication

Cell lines were not authenticated for this study

Mycoplasma contamination

All cell lines were tested negative for mycoplasma contamination

Commonly misidentified lines
(See ICLAC register)

No commonly misidentified cell lines were used.
